# Identification of Pulpitis-Related Potential Biomarkers Using Bioinformatics Approach

**DOI:** 10.1155/2021/1808361

**Published:** 2021-09-29

**Authors:** Bingchang Xin, Yuxiang Lin, He Tian, Jia Song, Liwei Zhang, Jian Lv

**Affiliations:** ^1^Department of Cariology and Endodontology, Qingdao Stomatological Hospital Affiliated to Qingdao University, 266001 Qingdao, Shandong Province, China; ^2^Department of Cariology and Endodontology 2nd, Stomatological Hospital of Xiamen Medical College, 361008 Xiamen, Fujian Province, China; ^3^Xiamen Key Laboratory of Stomatological Disease Diagnosis and Treatment, 361008 Xiamen, Fujian Province, China; ^4^Department of Dental Implant, Rizhao Stomatological Hospital, 276800 Rizhao, Shandong Province, China; ^5^Department of Preventive Dentistry, Qingdao Stomatological Hospital Affiliated to Qingdao University, 266001 Qingdao, Shandong Province, China

## Abstract

Inflammatory reaction of pulp tissue plays a role in the pathogen elimination and tissue repair. The evaluation of severity of pulpitis can serve an instructive function in therapeutic scheme. However, there are many limitations in the traditional evaluation methods for the severity of pulpitis. Based on the Gene Expression Omnibus (GEO) database, our study discovered 843 differentially expressed genes (DEGs) related to pulpitis. Afterwards, we constructed a protein-protein interaction (PPI) network of DEGs and used MCODE plugin to determine the key functional subset. Meanwhile, genes in the key functional subset were subjected to GO and KEGG enrichment analyses. The result showed that genes were mainly enriched in inflammatory reaction-related functions. Next, we screened out intersections of PPI network nodes and pulpitis-related genes. Then, 20 genes were obtained as seed genes. In the PPI network, 50 genes that had the highest correlation with seed genes were screened out using random walk with restart (RWR). Furthermore, 4 pulpitis-related hub genes were obtained from the intersection of the top 50 genes and genes in the key functional subset. Finally, GeneMANIA was utilized to predict genes coexpressed with hub genes, and expression levels of the 4 hub genes in normal and pulpitis groups were analyzed based on GEO data. The result demonstrated that the 4 hub genes were mainly coexpressed with chemokine-related genes and were remarkably upregulated in the pulpitis group. In short, we eventually determined 4 potential biomarkers of pulpitis.

## 1. Introduction

Pulpitis tissue is located in the dental pulp cavity composed of dentin, tooth enamel, and cementum, which can produce dentin, provide nutrition, and serve a supportive and protective role [1]. In most cases, pathogenic microorganism infection can cause pulpitis and treatments depend on disease severity. For instance, treatments on reversible pulpitis include direct pulp capping, indirect pulp capping, and pulp chamber pulpotomy, while irreversible pulpitis is usually treated with root canal therapy [2]. Hence, precise assessment on the severity of pulpitis is crucial to select appropriate therapy plans. Clinically, the assessment is mainly based on the degree of pain, pulp sensitivity test, and medical histories of patients, which is not effective and precise enough [2–4]. As a result, using pulpitis-related biomarkers to construct a precise evaluation scheme for pulpitis has become a hot research area.

Multiple studies on the detections of mRNA and protein expression levels revealed that various cytokines can serve as biomarkers of pulpitis [5]. In 2016, Mente et al. [6] pointed out that MMP-9 is expected to be a biomarker for the diagnosis of pulpitis with different degrees of severity. Recently, Chen et al. [7] found in their study that cells like CCL2, IL6, MMP9, and CXCL8 can be used as possible biomarkers of pulpitis. Based on current studies, it can be concluded that uncovering pulpitis-related biomarkers can provide foundation for the construction of new evaluation scheme for pulpitis.

In this study, based on pulpitis-related mRNA expression array from the Gene Expression Omnibus (GEO) database, we employed various analyses such as differential expression analysis, functional enrichment analysis, and protein-protein interaction (PPI) network analysis in this study. Through these efforts, 4 pulpitis-related hub genes were determined to be potential biomarkers of pulpitis, which bring a new insight to the improvements on the diagnostic scheme of pulpitis.

## 2. Methods

### 2.1. Data Preprocessing and Bioinformatics Analysis Flow Chart

**Figure 1:**
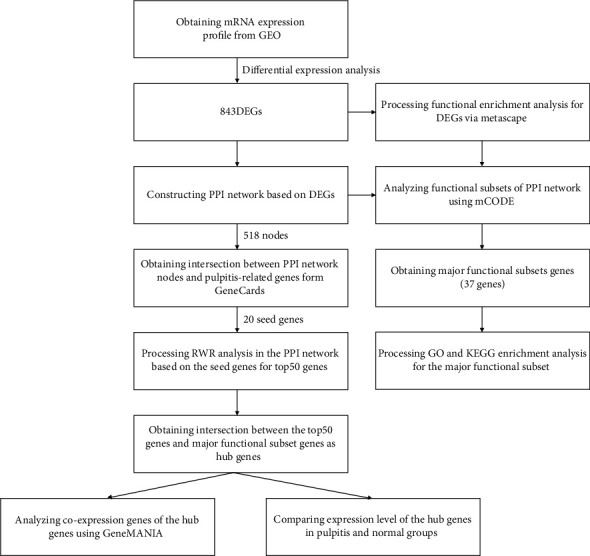
Bioinformatics analysis flow chart.

In this study, mRNA expression profile data from pulp tissue of 6 pulpitis patients and 6 healthy subjects were used for a series bioinformatics analyses. The dataset (GSE77459) was downloaded from the GEO database (https://www.ncbi.nlm.nih.gov/geo/), and the testing platform was GPL17692. Underlying the above data, we designed the following analysis procedures (Figure 1).

### 2.2. Differential Expression Analysis and Metascape Enrichment Analysis

With normal pulp tissue as the control, limma package [8] was utilized to differentially analyze the mRNA expression data of pulpitis tissue. Then, differentially expressed genes (DEGs) (∣logFC | >1, FDR < 0.05) were obtained. Furthermore, Metascape website (https://metascape.org/gp/index.html#/main/step1) was employed to analyze the functional enrichment of DEGs based on Reactome gene sets, canonical pathways, GO biological process, and KEGG pathway gene sets [9].

### 2.3. PPI Network Analysis

By utilizing the STRING database (https://string-db.org/cgi/input.pl?sessionId=kUBVwVTZIB02&input_page_active_form=multiple_identifiers), interaction score threshold was set as 0.7 and the PPI network of DEGs was constructed [10]. MCODE plugin [11] of Cytoscape software [12] was applied to screen out a key functional subset in the PPI network (parameter was set as default). R package clusterProfiler was used to perform GO and KEGG analyses on genes in the key functional subset (*q* value < 0.05) [13].

### 2.4. Pulpitis-Related DEGs Were Analyzed Using Random Walk with Restart (RWR) Algorithm

GeneCards (https://www.genecards.org/) was used to obtain pulpitis-related genes which were then intersected with node genes in the PPI network. The genes in the intersection were used as seed genes. Next, dnet package was utilized to conduct RWR algorithm analysis [14], and the restart probability was set as 0.85. In the PPI network, RWR algorithm was utilized to rank other genes based on given seed genes. In brief, the RWR algorithm was performed in a way of random walk from given nodes (seed genes) to adjacent nodes for several iterations, and a certain probability was set for returning to the starting nodes. After certain iterations, affinity score of each gene and seed genes was calculated, which was the basis for ranking the genes. Finally, the genes with an affinity score at the top 50 were selected for later analysis.

### 2.5. Expression and Coexpressed Genes of Pulpitis-Related Hub Genes

The top 50 genes obtained through the RWR algorithm were intersected with genes in the key functional subset from the PPI network, and the intersection genes were then used as pulpitis-related hub genes. GeneMANIA (http://genemania.org/) website tool was employed to search the coexpressed genes of hub genes, and their biological functions were analyzed [15]. Based on GEO-derived data, expression levels of hub genes in normal and pulpitis groups were analyzed. Differential expression was detected using the Wilcoxon test.

## 3. Results

### 3.1. Pulpitis-Related DEGs Are Determined, and Functional Enrichment Is Performed

**Figure 2:**
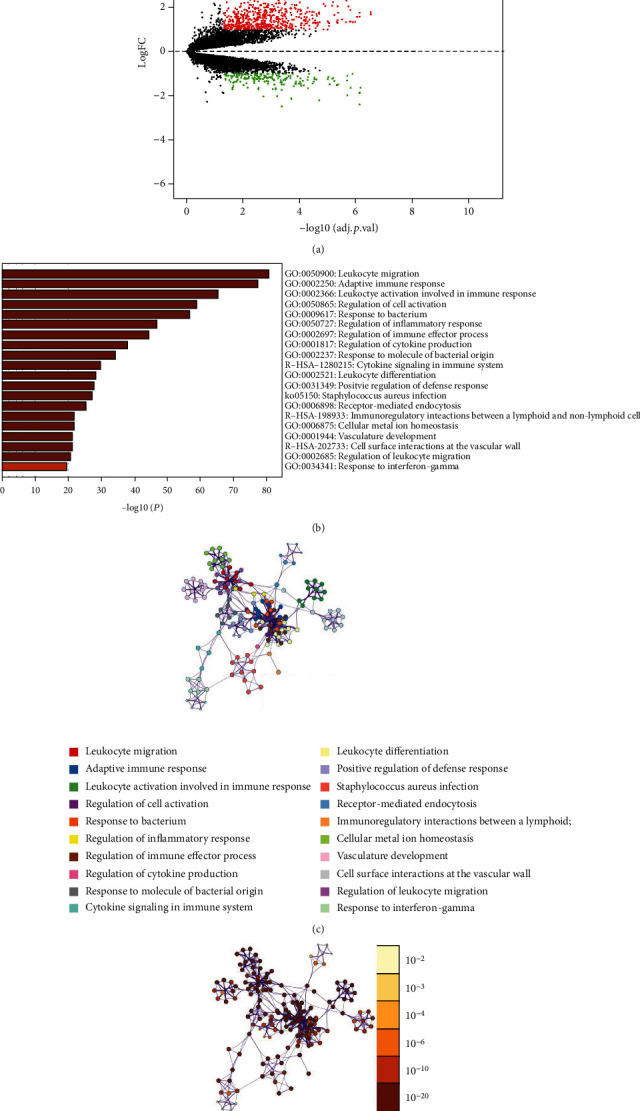
Differential expression analysis and functional enrichment analysis. (a) Analysis on the differences of mRNA expression profiles between pulpitis and normal pulp tissue samples (red dots indicate markedly upregulated genes while green dots indicate dramatically downregulated genes). (b) Bar chart of functional enrichment analysis on DEGs (terms are ranked according to *p* value). (c). Functional enrichment analysis network was displayed according to terms, with different colors representing different terms. Nodes with the same color belong to the same term. A thicker line means higher similarity between nodes, and a bigger node indicates more genes in node. (d) Functional enrichment network was exhibited according to *p* value, with darker color indicating smaller *p* value.

To determine pulpitis-related DEGs, we obtained mRNA expression profiles of pulpitis and normal pulp tissue from the GEO database. With the normal group as the control, differential expression analysis was performed on the pulpitis group (∣logFC | >1, FDR < 0.05). The analysis outcome manifested that 843 DEGs were obtained, in which 660 genes were significantly upregulated while 183 genes were remarkably downregulated (Supplementary Table 1) (Figure 2(a)). Afterwards, we utilized Metascape to analyze the functional enrichment of DEGs. The result exhibited that DEGs were mainly enriched in leukocyte migration, regulation of inflammatory, and regulation of cytokine production (Figures 2(b)–2(d)). As a result, we speculated that pulpitis-related DEGs were mainly associated with the function of leukocyte recruitment during inflammation.

### 3.2. PPI Network of DEGs Is Constructed

**Figure 3:**
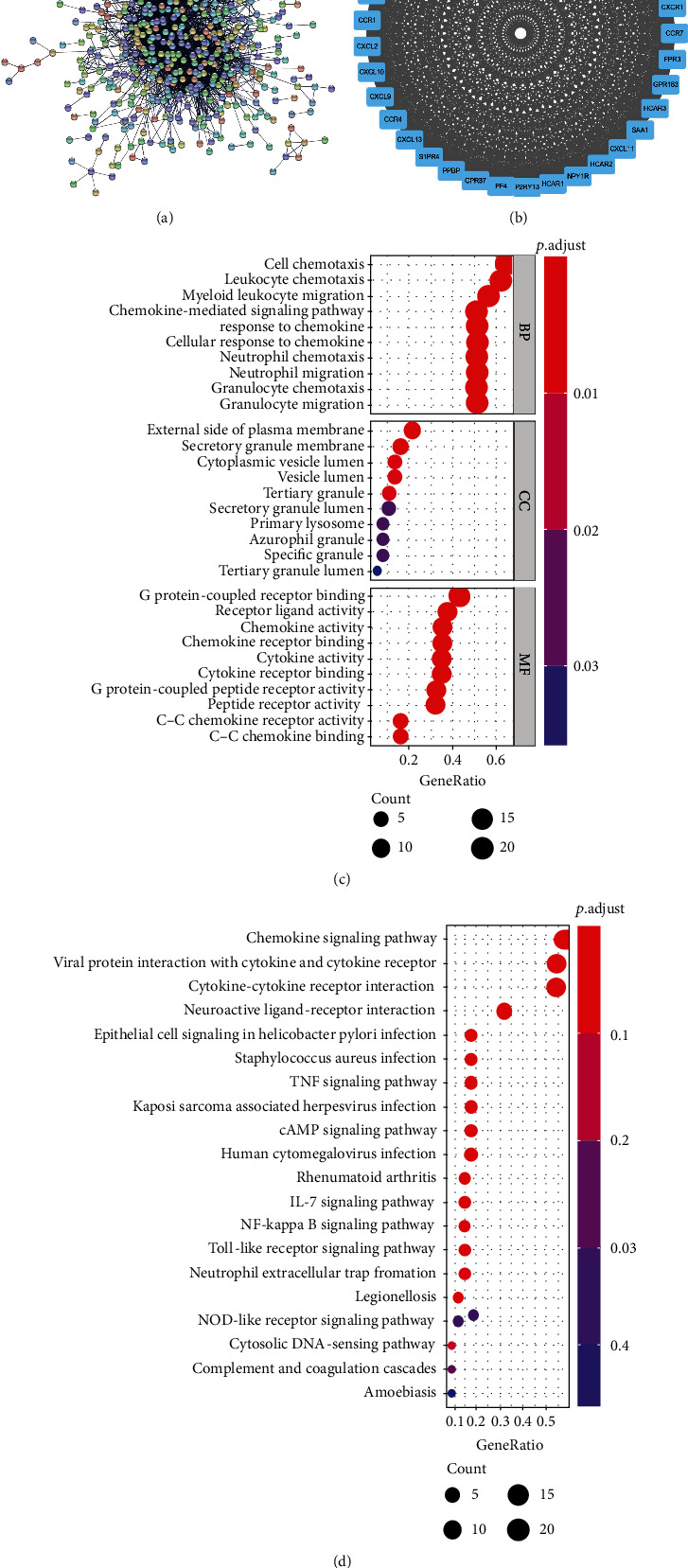
The construction of the PPI Network: (a). PPI network based on DEGs; (b) key functional subsets in PPI network; (c) bubble plot of GO enrichment analysis on genes in the key functional subset (the redder the color of the bubbles, the smaller the *p* value; the bigger the bubbles, the more genes enriched in pathways); (d) bubble plot of KEGG enrichment analysis on the genes in the key functional subset.

To explore the interaction between DEGs and corresponding proteins, we employed the STRING database to construct a PPI network of DEGs, which included 518 nodes and 3,322 lines (Figure 3(a)). MCODE plugin of Cytoscape software was used to analyze functional subsets in the PPI network from which the key functional subset composed of 37 genes was screened out (Supplementary Table 2) (Figure 3(b)). Thereafter, we performed GO and KEGG enrichment analyses to further understand the biological functions and signaling pathways in which the genes in the key functional subset were involved. The result of GO enrichment analysis showed that these genes were mainly enriched in functions like myeloid leukocyte migration, response to chemokine, and G protein-coupled receptor binding (Figure 3(c)). Meanwhile, the outcome of KEGG enrichment analysis demonstrated that the above genes were mainly gathered in chemokine, TNF and IL-17 signaling pathways (Figure 3(d)). All in all, the above results exhibited that the key functional subset in the PPI network may be related to the chemotactic effects on leucocytes in inflammatory reaction.

### 3.3. Pulpitis-Related Hub Genes Are Determined

**Figure 4:**
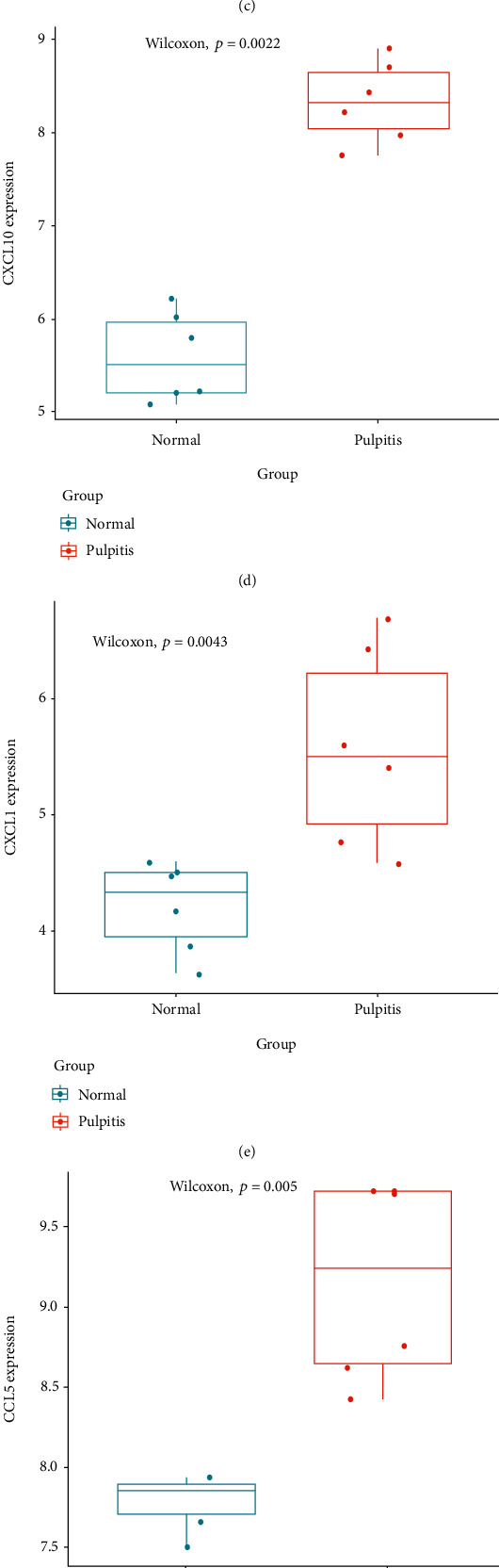
The determination of pulpitis-related hub genes. (a) Venn diagram about node genes in PPI network and pulpitis-related genes. (b) Venn diagram of genes with RWR-analyzed affinity score at the top 50 and genes in key functional subset. (c) GeneMANIA database was employed to analyze the gene-gene interaction network (nodes in the inner circle represent hub genes while nodes in the outer circle indicate genes coexpressed with hub genes. Purple lines represent coexpression relationship, and the colors of the nodes indicate the biological functions in which the corresponding genes were enriched). (d–g) Expression of hub genes (CXCL10, CXCL1, CCL5, and CXCR4) in the pulpitis and normal pulp groups.

To screen out the pulpitis-related hub genes, we firstly chose the intersection between 518 node genes in the PPI network and pulpitis-related genes (Supplementary Table 3) from GeneCards. Then, 20 genes were acquired (Supplementary Table 4) (Figure 4(a)). Afterwards, we used the 20 genes above as seed genes in the PPI network and employed the RWR algorithm to calculate the affinity score of each gene. The genes with an affinity score at the top 50 were reserved. Next, the 50 genes above were intersected with 37 genes in the key functional subset. Four genes were obtained to serve as pulpitis-related hub genes (Figure 4(b)). To explore the regulatory mechanism of hub genes in the occurrence of pulpitis, we applied GeneMANIA website tool to analyze the hub genes. The result manifested that a total of 20 genes had coexpression relationship with hub genes, which included chemotactic factors (CFs) of CXC families such as CXCL11, CXCL9, and CXCL8. Besides, as shown in Figure 4(c), these genes were also involved in the regulation of biological functions like chemokine activity, cytokine activity, and leukocyte migration. Finally, we drew a box plot based on GEO-derived data to confirm the expression levels of hub genes in pulpitis samples and normal samples. The result indicated that, compared with normal group, all of the 4 hub genes in the pulpitis group were dramatically upregulated (Figures 4(d)–4(g)). To sum up, hub genes were upregulated in pulpitis and may cooperate with other CFs to play a vital role in the pathogenetic process of pulpitis.

## 4. Discussion

During the pulpitis reaction, various cells (dentin cell, immune cell, and vascular endothelial cell) in pulpitis tissue secrete a great deal of inflammatory factors, including cell factor, CF, and neuropeptide [16]. These factors play significant roles in the reaction [16]. Through cell assay and animal assay, multiple studies also verified the expression and action mechanism of specific cell factor in pulpitis. For instance, the study by Li et al. [17] indicated the decrease in serum melatonin level of mice with acute pulpitis, which upregulates IL-1*β*, TLR4, and TNF-*α* expressions and activates the regulatory axis of TLR4/NK-*κ*B, accelerating inflammatory reaction. In addition, He et al. [18] used C57BL/6 mice to construct stable mouse models with pulpitis and observed changes on expression of IL-1*β*, IL-6, and TNF-*α* within 72 h in the mouse models. In our study, the result of Metascape functional enrichment analysis also manifested that pulpitis-related DEGs were remarkably gathered in regulation of cytokine production. This result held an agreement with the outcomes of the two studies above.

Although current studies have indicated the relationships between pulpitis and cell factors like interleukin, TNF-*α*, and NK-*κ*B, the related studies on the effect of CFs on pulpitis are relatively less. In our study, all of the 4 determined hub genes (CXCL10, CXCL1, CCL5, and CXCR4) were CFs, and many studies have illustrated the expression and action mechanism of these CFs in inflammatory reaction in different sites. Studies on the inflammatory reactions in the lung and liver demonstrated that CXCL10 and CCL5 are upregulated in inflammatory tissue and facilitate the inflammation and tissue fibrosis [19–21]. Additionally, another study based on mouse models with inflammation demonstrated that macrophage and mastocyte secrete CXCL1 which promotes the neutrophil recruitment in the early stage of inflammation [22]. Furthermore, chemokine CXCR4 functions as a proinflammatory factor in autoimmune diseases and inflammatory diseases, and suppression on its expression is considered to be a candidate plan for the treatment of such diseases [23–25]. In a word, the above study results confirmed that hub genes function as proinflammatory factors to play important roles in various inflammatory reactions. Similarly, hub genes obtained by the analysis in our study were upregulated in pulpitis patients.

The finally identified hub genes were all chemokines. GeneMANIA prediction also discovered a coexpression relationship between hub genes and other chemokine genes. Thus, chemokines are crucial for pulpitis. Meanwhile, genes in major functional subsets of the PPI network were mainly enriched in myeloid leukocyte migration. Some chemokines can stimulate inflammation and induce cells in the immune system at the infection sites during immune reaction [26]. This coincides with our results. Thus, we hypothesized that there may be abundant immune cell recruitments by regulation of chemokines during pulpitis inflammation.

In conclusion, our study downloaded mRNA expression profile data of pulpitis patients from the GEO database and obtained pulpitis-related DEGs by analyzing the data. Next, we performed functional enrichment and PPI network analyses to predict the DEG-related biological functions and key functional subsets in the PPI network. Afterwards, we employed the RWR algorithm to screen out the gene set that is closely associated with pulpitis in the PPI network. Besides, we also screened out the intersection between the gene set and key functional subset. Finally, 4 pulpitis-related hub genes were determined. Though the underlying biomarkers of pulpitis have been uncovered successfully using bioinformatics analysis, the study still needs to be improved. For instance, this study did not utilize approaches like molecular assay, cellular assay, and animal assay to deeply investigate the determined hub genes. Our next step, therefore, is to construct pulpitis mouse models and further study the expression of hub genes in pulpitis tissue of mouse models as well as the effects of the genes on inflammation.

## Data Availability

The data that support the findings of this study are available from the corresponding author upon reasonable request.

## Supplementary Materials

Supplementary 1 Supplementary Table 1: differentially expressed genes in the pulpitis group compared with those in the normal pulp group.

Supplementary 2 Supplementary Table 2: genes in the key functional subset.

Supplementary 3 Supplementary Table 3: pulpitis-related genes from GeneCards.

Supplementary 4 Supplementary Table 4: genes obtained by the intersection between pulpitis-related genes from GeneCards and genes in the key functional subset in the PPI network.
