# Supplementary material for: Identification of Pulpitis-Related Potential Biomarkers Using Bioinformatics Approach
Source: Comput Math Methods Med. 2021 Sep 29;2021:1808361. doi: 10.1155/2021/1808361 (PMC8495466; doi:10.1155/2021/1808361)
Supplement: Supplementary 1 — Supplementary Table 1: differentially expressed genes in the pulpitis group compared with those in the normal pulp group. [file 1808361.f1.pdf]

Gene	logFC	AveExpr	t	P.Value	adj.P.Val	B
PI3	4.755507	8.9144	43.94513	9.03E-16	1.65E-11	22.81231
CCL21	4.765654	6.88797	25.36917	1.22E-12	9.01E-09	18.38087
FOSB	2.4098	6.255286	24.94141	1.53E-12	9.01E-09	18.21396
IGKV1-5	6.690281	6.888127	24.45095	1.98E-12	9.01E-09	18.0171
PTGS2	2.48716	8.827145	21.36671	1.14E-11	4.14E-08	16.62937
SELE	3.342168	6.71415	20.65748	1.76E-11	5.34E-08	16.26917
SOCS3	2.802305	6.965161	19.78802	3.06E-11	7.96E-08	15.80372
ATP6V0D2	2.478125	3.892856	18.72628	6.21E-11	1.42E-07	15.19679
MMP9	4.685962	7.825304	17.37187	1.62E-10	2.91E-07	14.35456
CH25H	2.641359	5.40516	17.31703	1.69E-10	2.91E-07	14.31873
SLC37A2	1.794532	5.802078	17.16335	1.89E-10	2.91E-07	14.21755
PALMD	1.69833	8.070222	17.1468	1.92E-10	2.91E-07	14.20659
ACP5	3.014076	6.51626	16.32291	3.58E-10	5.03E-07	13.64387
IGHV1-3	4.38387	5.544778	16.00855	4.58E-10	5.58E-07	13.42005
CHI3L2	3.439598	6.196473	16.00721	4.59E-10	5.58E-07	13.41909
MOXD1	-1.65028	8.674568	-15.6847	5.94E-10	6.77E-07	13.18399
ACSL1	1.58766	7.768702	15.4578	7.14E-10	7.29E-07	13.01509
IGFBP4	1.768014	8.397571	15.37652	7.63E-10	7.29E-07	12.9539
IGHV3-72	6.670744	6.551916	15.32056	7.99E-10	7.29E-07	12.91155
SPON1	-1.89319	6.634197	-15.2886	8.20E-10	7.29E-07	12.88732
ADAMTS1	1.58432	5.667071	15.24884	8.47E-10	7.29E-07	12.85701
CRISPLD1	-1.86641	7.401258	-15.158	9.14E-10	7.29E-07	12.7875
NR1D1	-2.40474	7.286218	-15.1508	9.19E-10	7.29E-07	12.78196
ECSCR	1.582179	7.871135	14.85791	1.18E-09	8.93E-07	12.55445
PTPRB	1.350587	6.78332	14.62542	1.43E-09	1.04E-06	12.37017
CALCRL	1.338007	7.886592	14.37234	1.78E-09	1.25E-06	12.16579
KIT	-1.01932	10.18103	-14.2922	1.91E-09	1.29E-06	12.10021
ICAM2	1.368548	7.278305	14.03701	2.40E-09	1.51E-06	11.88871
DBP	-1.65676	6.028187	-14.0341	2.40E-09	1.51E-06	11.88624
LGALS2	1.889396	6.025192	13.98324	2.51E-09	1.53E-06	11.8436
ELTD1	1.370434	9.385103	13.89346	2.72E-09	1.60E-06	11.76785
NAMPT	2.052853	9.781379	13.73257	3.15E-09	1.79E-06	11.63076
GAP43	-1.99113	8.309238	-13.556	3.70E-09	1.96E-06	11.47827
EGR1	2.496983	9.049977	13.55293	3.71E-09	1.96E-06	11.47562
COL21A1	-1.10784	6.234225	-13.5356	3.77E-09	1.96E-06	11.46058
GHR	-1.3184	5.308193	-13.474	3.99E-09	2.02E-06	11.40674
AHSP	2.316066	6.202095	13.38727	4.32E-09	2.13E-06	11.33057
PLVAP	1.335958	7.517805	13.29914	4.69E-09	2.21E-06	11.25261
PPBP	2.360352	9.079336	13.29212	4.73E-09	2.21E-06	11.24638
COL4A1	1.464986	6.172029	12.9224	6.70E-09	3.05E-06	10.91308
NR1D2	-1.49802	7.790655	-12.5468	9.64E-09	4.21E-06	10.56406
CDH5	1.762801	7.006319	12.54061	9.70E-09	4.21E-06	10.55824
IL3RA	1.43348	7.392262	12.46447	1.05E-08	4.38E-06	10.48613
GJA5	2.057608	6.203099	12.43716	1.07E-08	4.38E-06	10.46016
FBLN5	1.306748	6.379343	12.43156	1.08E-08	4.38E-06	10.45482
RSPO2	-1.53069	6.347554	-12.3353	1.19E-08	4.71E-06	10.36278
PNP	1.351569	5.393333	12.31208	1.22E-08	4.72E-06	10.34045
SULF2	1.667451	7.886048	12.09082	1.52E-08	5.73E-06	10.12564
SLC38A4	-1.34156	4.754538	-12.0767	1.54E-08	5.73E-06	10.11178
OLFML1	-1.20639	8.472199	-11.9538	1.75E-08	6.37E-06	9.990638
LAPTM5	2.247844	9.74427	11.93221	1.79E-08	6.38E-06	9.96919
ALS2CR11	-1.22978	5.936483	-11.8706	1.90E-08	6.67E-06	9.907869
SAA2	2.083031	4.374126	11.82779	1.99E-08	6.84E-06	9.865058
IGHM	4.830957	8.802764	11.75692	2.14E-08	7.22E-06	9.793864
G0S2	2.802348	7.806545	11.59556	2.53E-08	8.33E-06	9.630169
PER3	-1.48697	8.88161	-11.5851	2.56E-08	8.33E-06	9.619484
SLC25A37	1.168018	8.678444	11.49363	2.82E-08	8.90E-06	9.525598

TGM2	1.299734	5.93462	11.48954	2.83E-08	8.90E-06	9.521382
CXCL10	2.748021	6.956804	11.47212	2.88E-08	8.91E-06	9.50342
METRNL	1.326043	6.404305	11.41344	3.07E-08	9.32E-06	9.442701
ECM2	-1.16183	7.567043	-11.3934	3.13E-08	9.37E-06	9.421928
HLF	-1.25879	8.691075	-11.3238	3.38E-08	9.82E-06	9.349312
MECOM	1.377995	4.916231	11.31855	3.39E-08	9.82E-06	9.343867
RAMP2	1.000395	9.542132	11.27267	3.57E-08	1.00E-05	9.295788
FOS	3.334404	7.316472	11.2705	3.57E-08	1.00E-05	9.293511
MT1H	4.196713	6.187719	11.228	3.74E-08	1.03E-05	9.248801
IL6	2.071315	4.667875	11.06987	4.44E-08	1.21E-05	9.081013
JUNB	2.165273	8.852041	10.93352	5.15E-08	1.38E-05	8.934491
SLCO2A1	1.006281	9.08087	10.90695	5.31E-08	1.40E-05	8.905739
ACTA2	1.334219	5.576804	10.61114	7.38E-08	1.90E-05	8.581109
CALB1	-2.21466	6.451239	-10.5517	7.90E-08	2.00E-05	8.514895
NRGN	1.718623	5.581159	10.53113	8.08E-08	2.00E-05	8.491856
SRGN	1.783641	10.35145	10.52523	8.14E-08	2.00E-05	8.48525
NFIL3	1.244205	7.209025	10.44048	8.96E-08	2.18E-05	8.389981
ITPR3	1.323899	5.67944	10.39421	9.45E-08	2.27E-05	8.337669
S100A8	1.164959	10.80279	10.36797	9.74E-08	2.31E-05	8.307904
SLC7A5	2.634525	6.075008	10.3209	1.03E-07	2.39E-05	8.254349
EDN1	1.624937	5.629704	10.31332	1.04E-07	2.39E-05	8.245696
BCL3	1.712647	6.171129	10.27967	1.08E-07	2.46E-05	8.207248
MPP6	-1.26996	8.419667	-10.2573	1.11E-07	2.49E-05	8.181672
IER3	1.307906	8.729255	10.18853	1.20E-07	2.63E-05	8.102531
IFI30	2.508708	9.379735	10.16962	1.23E-07	2.63E-05	8.080692
KDR	1.065598	6.897461	10.1656	1.23E-07	2.63E-05	8.07605
RASGRP3	1.281229	5.667889	10.15918	1.24E-07	2.63E-05	8.068623
COL15A1	1.418651	5.920688	10.09428	1.34E-07	2.77E-05	7.993342
PER1	-1.188	6.939521	-10.0451	1.42E-07	2.90E-05	7.936007
EYA1	-1.25628	6.088186	-10.0349	1.43E-07	2.91E-05	7.924058
BTLA	1.799398	4.942409	9.971133	1.55E-07	3.06E-05	7.849306
TAGLN	1.32212	6.402618	9.963847	1.56E-07	3.06E-05	7.840734
MCTP1	1.796709	5.970824	9.95871	1.57E-07	3.06E-05	7.834687
EFNA5	-1.36244	7.411493	-9.95369	1.58E-07	3.06E-05	7.82878
GPR116	1.307929	7.058606	9.88903	1.71E-07	3.27E-05	7.752394
TNFRSF21	1.502775	6.894378	9.845812	1.80E-07	3.39E-05	7.701096
LRRTM2	-1.45302	6.945518	-9.84229	1.80E-07	3.39E-05	7.696905
IGLV2-11	3.589893	9.132312	9.826236	1.84E-07	3.42E-05	7.677794
SLC16A10	1.801883	4.625837	9.78733	1.93E-07	3.54E-05	7.631364
CCL2	1.655472	9.910571	9.780807	1.94E-07	3.54E-05	7.623563
INHBA	1.813672	6.34256	9.584179	2.46E-07	4.41E-05	7.38628
TREM1	2.986494	6.290516	9.573953	2.50E-07	4.41E-05	7.373826
ITGAX	3.284416	6.058498	9.562365	2.53E-07	4.41E-05	7.359697
SOX18	1.446008	6.007908	9.546534	2.58E-07	4.41E-05	7.340374
NR4A3	2.429588	3.891434	9.536675	2.61E-07	4.41E-05	7.328325
ANPEP	2.546554	6.4981	9.529868	2.63E-07	4.41E-05	7.32
SLC6A6	1.017739	7.516484	9.529866	2.63E-07	4.41E-05	7.319997
KIAA0040	1.178216	7.986373	9.511751	2.69E-07	4.46E-05	7.297818
UNC5C	-1.01975	6.256719	-9.49464	2.75E-07	4.52E-05	7.276831
RGN	-1.66752	5.837519	-9.39633	3.11E-07	5.00E-05	7.155648
TET1	-1.1673	7.313342	-9.39131	3.12E-07	5.00E-05	7.149433
PCDH17	1.988535	5.562007	9.372508	3.20E-07	5.07E-05	7.126119
GPR4	1.365804	6.467624	9.366075	3.22E-07	5.07E-05	7.118135
MUSK	-1.29277	4.230906	-9.34978	3.29E-07	5.13E-05	7.097888
IBSP	1.7999	9.652959	9.293621	3.53E-07	5.45E-05	7.027884
MLKL	1.558462	6.409509	9.277819	3.60E-07	5.49E-05	7.008122
FXYD5	1.278683	7.995601	9.274361	3.62E-07	5.49E-05	7.003793
SEMA6B	1.473687	5.382114	9.205496	3.94E-07	5.94E-05	6.917312

SGIP1	1.382366	4.99586	9.185299	4.04E-07	6.04E-05	6.891848
PARM1	1.730699	5.944331	9.170299	4.12E-07	6.07E-05	6.872904
AQP1	1.280038	7.089099	9.152026	4.22E-07	6.13E-05	6.849792
CHRDL2	1.408901	5.365831	9.148733	4.23E-07	6.13E-05	6.845623
IDO1	1.572495	3.774809	9.07843	4.63E-07	6.59E-05	6.756321
CADM3	1.191857	6.66689	9.054021	4.78E-07	6.75E-05	6.725181
KLF2	1.433905	7.089894	9.030841	4.92E-07	6.90E-05	6.695545
DPP4	1.043146	4.967314	9.015461	5.02E-07	6.98E-05	6.675847
C3	2.986682	6.790087	8.991092	5.18E-07	7.04E-05	6.644581
PIK3C2B	1.413131	5.794807	8.986173	5.21E-07	7.04E-05	6.638261
ANO5	-1.54367	7.197187	-8.98548	5.21E-07	7.04E-05	6.637376
ASIP	-1.33694	5.808999	-8.91419	5.71E-07	7.55E-05	6.545462
TM4SF1	1.291067	6.791807	8.847333	6.23E-07	8.17E-05	6.458713
S100A9	1.710545	10.30113	8.813655	6.51E-07	8.48E-05	6.414816
HAS2	1.518635	5.829653	8.789985	6.71E-07	8.64E-05	6.383884
TES	1.287323	5.593274	8.785152	6.76E-07	8.64E-05	6.377559
GREB1L	-1.55883	4.896189	-8.78289	6.78E-07	8.64E-05	6.374601
ENG	1.08293	8.211475	8.776229	6.83E-07	8.65E-05	6.365876
MPZ	1.20309	8.9083	8.745126	7.12E-07	8.90E-05	6.325077
ABCA1	1.868497	7.382412	8.744484	7.12E-07	8.90E-05	6.324233
CD84	1.850926	5.706377	8.723318	7.32E-07	9.01E-05	6.296402
EDIL3	-1.46366	8.485774	-8.71897	7.37E-07	9.01E-05	6.29068
IRF8	1.905632	6.247443	8.691332	7.64E-07	9.24E-05	6.254239
ATP1A2	-1.22582	7.189786	-8.69022	7.65E-07	9.24E-05	6.252765
SOD2	1.69697	6.368314	8.673073	7.82E-07	9.38E-05	6.230116
C14orf39	-1.36599	5.647371	-8.66479	7.91E-07	9.41E-05	6.219163
SDS	2.233465	4.541828	8.634869	8.23E-07	9.68E-05	6.179511
LST1	1.563111	6.591221	8.577894	8.87E-07	0.000103	6.103711
DPT	-1.4634	8.450884	-8.57501	8.91E-07	0.000103	6.099866
FPR1	2.411106	8.670006	8.547865	9.23E-07	0.000105	6.063602
IGHA2	3.607637	8.405286	8.522327	9.55E-07	0.000108	6.029404
MNDA	1.90898	7.843895	8.49786	9.87E-07	0.00011	5.996565
MAP2K6	-1.12164	7.477432	-8.45547	1.04E-06	0.000116	5.939491
LCP1	1.810193	8.787823	8.441437	1.06E-06	0.000118	5.920554
IGLV8-61	2.728773	5.54511	8.373702	1.17E-06	0.000126	5.828783
SFRP4	2.112316	5.947151	8.370984	1.17E-06	0.000126	5.825088
BIRC3	1.583155	6.636063	8.364113	1.18E-06	0.000127	5.815744
CSF3R	2.510648	6.222862	8.337697	1.22E-06	0.00013	5.779768
SYT1	-1.47315	6.788026	-8.32272	1.25E-06	0.000132	5.759325
BARX1	-1.37038	6.56663	-8.31456	1.26E-06	0.000132	5.748188
DUSP1	1.946568	7.575733	8.283186	1.32E-06	0.000137	5.705251
C10orf128	1.137439	5.427368	8.268832	1.34E-06	0.000138	5.685568
T6GALNAC	-1.46071	5.445139	-8.24575	1.39E-06	0.000141	5.653862
LRRC15	2.109796	7.898377	8.20037	1.48E-06	0.000146	5.591325
PTAFR	1.997518	4.631509	8.193492	1.49E-06	0.000147	5.581824
CD68	1.450175	10.39808	8.138715	1.61E-06	0.000157	5.505943
CHRDL1	-1.5776	7.612676	-8.1293	1.63E-06	0.000158	5.492863
IGHG4	2.995766	9.82101	8.125131	1.64E-06	0.000158	5.487067
ABCC9	1.023562	4.912683	8.078468	1.75E-06	0.000167	5.422043
C15orf48	4.166147	6.246356	8.03524	1.85E-06	0.000174	5.361555
MPPED2	-1.01753	6.227304	-8.0339	1.86E-06	0.000174	5.359671
IGHG3	3.623651	9.042413	8.023228	1.88E-06	0.000176	5.344705
CLDN5	1.16125	4.333596	8.019318	1.89E-06	0.000176	5.339215
FPR3	2.138423	7.119015	7.973581	2.02E-06	0.000182	5.274861
CLIC2	1.166833	6.605278	7.968584	2.03E-06	0.000182	5.267813
FAM129A	1.445104	6.426561	7.966991	2.04E-06	0.000182	5.265565
CCDC109E	1.236486	7.444094	7.966662	2.04E-06	0.000182	5.265101
ALAS2	2.60747	7.303532	7.951615	2.08E-06	0.000184	5.243856

PTGS1	1.099405	5.316173	7.93902	2.12E-06	0.000187	5.226052
CD34	1.009778	7.873185	7.933257	2.14E-06	0.000187	5.217898
COL10A1	1.670482	5.879802	7.910516	2.21E-06	0.000192	5.185678
SH3GL2	-2.15491	4.4695	-7.89581	2.25E-06	0.000194	5.164808
NABP1	1.961666	7.49066	7.892897	2.26E-06	0.000194	5.16067
LPPR5	-2.13403	6.222875	-7.88068	2.30E-06	0.000196	5.143304
HBQ1	1.766317	6.747742	7.847632	2.41E-06	0.000204	5.096236
WARS	1.071675	6.412933	7.820783	2.50E-06	0.000209	5.057889
IGLL5	1.96099	5.359876	7.817545	2.51E-06	0.000209	5.053258
SLC7A10	-1.20855	8.028718	-7.81161	2.54E-06	0.000209	5.044764
AIG1	-1.10681	6.078615	-7.80896	2.54E-06	0.000209	5.04098
HCLS1	1.980027	6.829038	7.807078	2.55E-06	0.000209	5.038279
NRIP3	1.261202	3.974201	7.806007	2.56E-06	0.000209	5.036746
CDA	1.172656	6.839614	7.766826	2.70E-06	0.00022	4.980539
CXCL2	2.510044	6.526521	7.763248	2.72E-06	0.00022	4.975395
GPR183	2.405337	7.880174	7.750937	2.76E-06	0.000222	4.957687
MYCT1	1.087187	7.960405	7.701218	2.97E-06	0.000234	4.885967
IGKV1-16	3.213113	9.179202	7.695025	2.99E-06	0.000234	4.877011
USP51	-1.1204	6.438449	-7.65181	3.18E-06	0.000245	4.814367
LRRC32	1.396219	6.237438	7.62999	3.29E-06	0.00025	4.782646
APLNR	1.303168	7.215576	7.574686	3.56E-06	0.000267	4.701956
PLK3	1.177685	5.587947	7.531104	3.79E-06	0.000283	4.638078
MT1G	3.420687	6.506935	7.526301	3.82E-06	0.000284	4.631022
CACNB4	-1.1896	4.948679	-7.52407	3.83E-06	0.000284	4.627741
CSTA	1.792684	6.914696	7.514728	3.88E-06	0.000286	4.61401
ARHGEF15	1.099466	5.724551	7.508323	3.92E-06	0.000288	4.604587
NR4A2	1.840457	6.066137	7.499676	3.97E-06	0.00029	4.591855
SPI1	1.503362	6.458835	7.495371	3.99E-06	0.000291	4.585514
PCSK2	-1.2392	6.194719	-7.47293	4.12E-06	0.000297	4.552418
IL33	1.116127	5.6347	7.459813	4.20E-06	0.0003	4.533035
LMO3	-1.01165	5.554522	-7.45752	4.22E-06	0.0003	4.529645
LYZ	1.934629	10.07878	7.446296	4.29E-06	0.000304	4.513042
CSF2RB	2.234636	6.20851	7.427855	4.40E-06	0.000308	4.485724
TOP2A	1.340411	4.132868	7.414716	4.49E-06	0.000312	4.466232
C1orf162	1.816878	6.901406	7.412591	4.50E-06	0.000312	4.463078
CD226	1.593082	5.186728	7.394281	4.63E-06	0.000318	4.435869
PEX11A	-1.23466	6.231096	-7.39121	4.65E-06	0.000318	4.431295
IGHA1	3.466832	9.202559	7.389325	4.66E-06	0.000318	4.428498
CASQ2	1.210717	5.084988	7.389282	4.66E-06	0.000318	4.428433
LILRA5	2.193358	5.282363	7.381558	4.71E-06	0.00032	4.416937
SPP1	1.920084	9.450584	7.380976	4.72E-06	0.00032	4.416071
LMNB1	1.022343	5.69205	7.375519	4.76E-06	0.000321	4.407943
LECT1	-1.2962	4.890846	-7.36781	4.81E-06	0.000323	4.396453
SQRDL	1.707989	7.5723	7.367525	4.81E-06	0.000323	4.39603
GJA4	1.375319	5.688415	7.359556	4.87E-06	0.000325	4.384145
EMR2	2.364858	6.428591	7.322251	5.14E-06	0.000341	4.328394
PROX1	1.166582	3.780222	7.318581	5.17E-06	0.000341	4.322899
SLC11A1	2.148911	5.536995	7.316912	5.18E-06	0.000341	4.320399
FGL2	1.654085	8.440633	7.30458	5.28E-06	0.000346	4.301918
PIK3R5	1.699471	5.281234	7.289407	5.40E-06	0.000353	4.279151
TRHDE	-1.49847	3.316531	-7.281	5.47E-06	0.000356	4.26652
FAM196B	-1.00894	4.838914	-7.27284	5.53E-06	0.000358	4.254262
CD93	1.317669	6.365137	7.244508	5.77E-06	0.000369	4.211595
CLEC2B	1.374791	6.117041	7.231492	5.88E-06	0.000374	4.19196
OSR2	-1.29838	5.477894	-7.23041	5.89E-06	0.000374	4.190322
TRIB1	1.609489	5.329932	7.180698	6.35E-06	0.000396	4.115107
CMTM2	1.493585	5.249839	7.179287	6.36E-06	0.000396	4.112968
PTPRE	1.378775	6.579637	7.177108	6.38E-06	0.000396	4.109662

IL6R	1.203619	5.479499	7.172685	6.42E-06	0.000396	4.102952
CCL22	1.247078	6.310996	7.150116	6.64E-06	0.000404	4.068664
CCL23	1.755816	4.620731	7.12431	6.91E-06	0.000416	4.029373
TFPI2	1.973058	5.136514	7.122982	6.92E-06	0.000416	4.027348
CLEC4E	2.483727	5.003335	7.109067	7.07E-06	0.000423	4.006122
SCO2	1.060285	6.417981	7.107208	7.09E-06	0.000423	4.003283
SLC38A11	-2.49474	6.171944	-7.10515	7.11E-06	0.000423	4.000142
GLIPR1L2	-1.39239	5.313723	-7.10444	7.12E-06	0.000423	3.999061
LIX1	-1.74396	4.451663	-7.09507	7.22E-06	0.000427	3.984741
BMP5	-1.49899	4.998286	-7.09079	7.26E-06	0.000427	3.978204
ZFP36	1.643442	9.970575	7.07662	7.42E-06	0.000435	3.956519
DKK2	1.2715	5.146823	7.036948	7.88E-06	0.000456	3.895671
SLC6A1	-1.30038	4.865056	-7.03472	7.91E-06	0.000456	3.892247
GLRX	1.110358	4.726356	7.031402	7.95E-06	0.000456	3.887148
CORO1A	1.884963	6.426606	7.02562	8.02E-06	0.000458	3.878257
GRIA1	-1.41099	3.856486	-6.99944	8.34E-06	0.000474	3.837939
CFI	1.464811	7.626664	6.988883	8.47E-06	0.00048	3.821658
COL25A1	-1.13964	5.572305	-6.9508	8.98E-06	0.0005	3.762792
FCER1G	1.472175	10.11509	6.945465	9.05E-06	0.0005	3.754521
SCD	1.056098	8.338415	6.944696	9.06E-06	0.0005	3.75333
CXCR4	2.19744	8.278742	6.9405	9.12E-06	0.0005	3.746828
CD300A	1.863495	5.554505	6.901201	9.69E-06	0.000526	3.685803
AQP9	3.659878	5.944476	6.891754	9.83E-06	0.000532	3.671103
IGLV1-40	3.847878	7.537996	6.8863	9.91E-06	0.000533	3.662609
ALOX5AP	1.782204	8.318757	6.884329	9.94E-06	0.000533	3.659539
LPHN3	-1.00315	4.746114	-6.88306	9.96E-06	0.000533	3.657557
CLEC7A	2.202698	6.25614	6.877013	1.01E-05	0.000536	3.648137
SELP	1.367501	5.781462	6.860644	1.03E-05	0.000546	3.6226
C5AR1	2.310652	7.268657	6.853488	1.04E-05	0.000549	3.611424
CFB	1.453275	5.679977	6.847782	1.05E-05	0.000553	3.602508
WDFY4	1.5022	4.977986	6.842149	1.06E-05	0.000556	3.5937
SYT2	-1.10928	6.403496	-6.83916	1.07E-05	0.000556	3.589019
CSRNP1	1.360985	4.991528	6.837868	1.07E-05	0.000556	3.587004
IL2RG	2.559692	6.644547	6.831064	1.08E-05	0.00056	3.576355
ANGPT2	1.065641	6.520423	6.826929	1.09E-05	0.000562	3.569882
CLEC4A	1.199226	6.126933	6.812715	1.11E-05	0.000566	3.547609
C10orf10	1.310558	7.080986	6.807108	1.12E-05	0.000569	3.538814
SELL	2.016732	6.127258	6.794347	1.14E-05	0.000577	3.518783
CHRM2	-1.2209	5.203195	-6.78895	1.15E-05	0.00058	3.510309
LAT	1.581937	6.519979	6.785767	1.16E-05	0.000581	3.505302
LAMB1	1.070328	7.25067	6.761494	1.20E-05	0.000598	3.467107
GIMAP8	1.170015	6.632816	6.754014	1.22E-05	0.000604	3.455319
HIST1H4D	-2.02912	7.330049	-6.73727	1.25E-05	0.000618	3.42891
ADCY4	1.105472	5.678449	6.718096	1.29E-05	0.000626	3.398609
ITGA11	1.479367	5.095402	6.714786	1.29E-05	0.000626	3.393375
USH1C	-1.09467	5.389921	-6.70487	1.31E-05	0.000634	3.377678
GKV3D-1[illegible]	3.262613	9.187015	6.692483	1.34E-05	0.000643	3.358059
TNFRSF10[illegible]	1.567126	5.525885	6.690269	1.34E-05	0.000644	3.354548
PLAUR	2.186306	9.089063	6.670628	1.38E-05	0.000659	3.323384
NCF2	2.182438	7.025772	6.646071	1.44E-05	0.000677	3.28434
FCGR2A	1.377818	5.092168	6.642778	1.45E-05	0.000677	3.279097
FAP	1.520564	4.365853	6.639955	1.45E-05	0.000679	3.274603
CDH12	-1.01321	8.455812	-6.62083	1.50E-05	0.000692	3.24412
SLC10A4	-1.66875	4.734288	-6.61879	1.50E-05	0.000693	3.240863
MCAM	1.148278	5.098077	6.611414	1.52E-05	0.000698	3.229092
CNTN3	-1.71798	5.398885	-6.61077	1.52E-05	0.000698	3.228066
TC2N	1.862379	5.532483	6.602948	1.54E-05	0.000704	3.215571
PTPRC	2.487536	7.781753	6.549516	1.67E-05	0.000755	3.129995

APOBEC3A	1.949286	4.691111	6.544729	1.69E-05	0.000757	3.122308
CLEC14A	1.048463	5.881042	6.526974	1.73E-05	0.000769	3.09377
LRRC17	-1.3014	5.868618	-6.52384	1.74E-05	0.00077	3.088727
GSTA1	-1.50155	5.153904	-6.52336	1.74E-05	0.00077	3.087954
EMR3	1.931056	5.094722	6.518387	1.76E-05	0.000771	3.079951
LRG1	1.340118	5.701679	6.493852	1.83E-05	0.000795	3.04041
SIRPA	1.655249	6.948694	6.48398	1.86E-05	0.0008	3.024478
TRBC2	2.054901	9.175758	6.483976	1.86E-05	0.0008	3.024471
ATP1B2	-1.30363	8.512526	-6.48173	1.86E-05	0.000801	3.020851
VWF	1.409419	7.884508	6.471607	1.89E-05	0.000807	3.004487
OGN	-1.29537	9.182715	-6.44545	1.97E-05	0.000833	2.962149
GADD45B	1.655508	8.411723	6.440258	1.99E-05	0.000837	2.953741
OASL	1.074465	6.538664	6.427203	2.03E-05	0.000846	2.932567
HLA-DMA	1.225498	6.877957	6.404526	2.11E-05	0.000867	2.895728
CST7	1.892524	7.42524	6.401952	2.12E-05	0.000868	2.891542
DUT	-1.01048	4.759719	-6.39535	2.14E-05	0.00087	2.880803
B4GALT6	-1.70947	7.041616	-6.38002	2.19E-05	0.000887	2.855843
NNMT	1.640459	5.156136	6.371342	2.22E-05	0.000894	2.841688
PTPN22	1.892914	4.871146	6.359619	2.26E-05	0.000905	2.822561
CHI3L1	1.854995	5.338219	6.358648	2.27E-05	0.000905	2.820975
THRA	-1.23618	7.900379	-6.35835	2.27E-05	0.000905	2.820496
ASIC3	-1.06595	6.400286	-6.34885	2.30E-05	0.000915	2.804967
CCR7	1.799194	6.266266	6.335799	2.35E-05	0.000927	2.783633
HLA-DQA2	1.924529	8.230528	6.328436	2.38E-05	0.000931	2.771585
HCK	2.135314	6.760919	6.326849	2.39E-05	0.000932	2.768986
MAP3K8	1.105693	6.372096	6.324358	2.40E-05	0.000934	2.764907
FAM154B	-1.15432	4.261406	-6.30375	2.48E-05	0.000955	2.731123
GIMAP4	1.086935	8.21912	6.276381	2.59E-05	0.000985	2.686178
FMNL1	1.339652	5.98628	6.269529	2.62E-05	0.000992	2.674906
LAIR1	1.142477	4.430064	6.265333	2.64E-05	0.000997	2.668001
S1PR4	1.608709	5.803354	6.25768	2.67E-05	0.001007	2.655401
VGLL2	1.872055	5.757273	6.254907	2.68E-05	0.00101	2.650832
PLAC8	1.540033	5.473236	6.253489	2.69E-05	0.00101	2.648496
HLA-A	1.163689	6.385233	6.244664	2.73E-05	0.001014	2.633949
PRKAA2	-1.19439	4.651665	-6.23642	2.76E-05	0.001026	2.620354
CHST15	1.504442	6.386651	6.223279	2.82E-05	0.001046	2.598654
GKV3D-11	3.328241	6.855727	6.218901	2.84E-05	0.001051	2.59142
EFHD2	1.212357	7.353668	6.215908	2.86E-05	0.001053	2.586473
ACSL5	1.471242	5.80256	6.215287	2.86E-05	0.001053	2.585446
GJC3	1.201514	6.139133	6.213839	2.87E-05	0.001053	2.583053
GCA	1.030812	6.460178	6.197199	2.94E-05	0.001078	2.55552
CIITA	1.932822	5.866523	6.184963	3.00E-05	0.001095	2.53525
BCL2A1	3.24242	6.471608	6.181707	3.02E-05	0.001099	2.529852
VNN2	1.783785	5.91549	6.177494	3.04E-05	0.001104	2.522866
IL1R2	1.118617	5.098586	6.173699	3.06E-05	0.001109	2.516571
ARHGAP30	1.399159	5.737174	6.171082	3.07E-05	0.001109	2.512229
CD44	1.396969	6.095309	6.166056	3.10E-05	0.00111	2.503886
FIGNL1	-1.16843	4.824837	-6.15363	3.16E-05	0.001129	2.483238
F11R	1.669	6.48689	6.147472	3.19E-05	0.001135	2.473009
FAM49B	1.01681	6.576011	6.147022	3.19E-05	0.001135	2.47226
LYN	1.636995	7.595978	6.119163	3.34E-05	0.001174	2.425878
ICAM1	1.646142	5.792953	6.118327	3.35E-05	0.001174	2.424484
TIMP4	1.251092	6.665309	6.115107	3.37E-05	0.001178	2.419115
TNFSF10	1.197843	7.839739	6.091119	3.50E-05	0.00122	2.379075
ATF3	1.150156	4.565714	6.077826	3.58E-05	0.001243	2.35685
BEND6	-1.02096	6.371845	-6.0635	3.66E-05	0.001256	2.332864
CYR61	2.105966	6.909359	6.051792	3.73E-05	0.001278	2.313252
HMOX1	1.181628	7.346093	6.040337	3.81E-05	0.001287	2.294039

TOX3	-1.18157	5.189689	-6.03599	3.83E-05	0.001291	2.286742
SEMA3D	-1.35827	4.84256	-6.0349	3.84E-05	0.001291	2.284918
CD37	2.161701	7.675596	6.03418	3.84E-05	0.001291	2.283705
LCTL	-1.46198	5.215774	-6.02482	3.90E-05	0.001309	2.267986
WAS	2.007941	6.008995	6.02095	3.93E-05	0.001315	2.261478
HAPLN3	1.215167	5.3434	6.01488	3.97E-05	0.001323	2.251273
HLA-DMB	1.653738	7.258271	5.995397	4.10E-05	0.001364	2.218481
TEK	1.072253	5.652423	5.985224	4.17E-05	0.001377	2.201335
DPP6	-1.21695	5.499007	-5.98402	4.18E-05	0.001377	2.199298
TACC3	1.022981	4.777898	5.980264	4.20E-05	0.001383	2.192971
ZNF365	-1.0208	3.784521	-5.97164	4.26E-05	0.001398	2.178414
CD53	2.069378	8.469412	5.966681	4.30E-05	0.001405	2.170048
TMEM154	1.239895	5.835457	5.956362	4.37E-05	0.001426	2.152615
CD1C	1.163288	4.836452	5.950991	4.41E-05	0.001434	2.143537
CNTN4	-1.20322	7.110185	-5.94062	4.49E-05	0.00145	2.125996
EVI2B	1.777739	7.490641	5.940158	4.49E-05	0.00145	2.125211
IGKV1-6	3.659676	7.861554	5.93156	4.56E-05	0.001467	2.110654
LIF	1.072154	4.636142	5.921694	4.63E-05	0.001485	2.093938
NCKAP1L	1.850641	7.150797	5.918388	4.66E-05	0.00149	2.088334
EFCAB1	-1.3358	5.008137	-5.90603	4.76E-05	0.001503	2.067365
CTSS	2.013974	8.913748	5.897434	4.82E-05	0.001511	2.052776
AMICA1	1.500572	5.822265	5.887872	4.90E-05	0.001528	2.036529
SLAMF7	2.55719	5.608549	5.881002	4.96E-05	0.001539	2.024848
OSMR	1.187774	7.8202	5.880822	4.96E-05	0.001539	2.024541
LILRB1	1.210729	4.962419	5.880398	4.96E-05	0.001539	2.023822
KCNH5	-1.13281	4.350822	-5.87582	5.00E-05	0.001548	2.016041
TLR2	1.715714	5.381397	5.873542	5.02E-05	0.001551	2.012156
PXDN	1.12321	5.248582	5.867226	5.07E-05	0.00156	2.001405
CXCL9	1.883815	7.054167	5.857142	5.16E-05	0.001579	1.984227
IGLV3-19	3.537563	5.524006	5.849354	5.23E-05	0.001597	1.970951
RASD1	1.5909	6.149434	5.848326	5.24E-05	0.001597	1.969199
APBB1IP	2.007918	5.472918	5.844761	5.27E-05	0.001601	1.963117
TP63	-1.14628	4.832801	-5.83977	5.31E-05	0.001606	1.954598
XCR1	1.157652	4.947865	5.839494	5.31E-05	0.001606	1.954131
CXCR1	2.176622	5.813128	5.83893	5.32E-05	0.001606	1.953168
LRRN3	-1.11635	4.504272	-5.83168	5.38E-05	0.00162	1.940798
IGKV3D-7	2.773634	8.721246	5.818563	5.50E-05	0.001645	1.918378
FAM46C	1.670518	8.338036	5.805151	5.63E-05	0.001672	1.895437
COL14A1	1.409979	6.259647	5.79816	5.70E-05	0.001683	1.883469
ATP2A3	1.297881	5.419681	5.79223	5.75E-05	0.00169	1.873311
CD86	1.819367	6.810474	5.788626	5.79E-05	0.001697	1.867135
SLPI	1.803335	8.128693	5.783341	5.84E-05	0.001709	1.858075
BIN2	1.880778	6.175219	5.779613	5.88E-05	0.001715	1.851683
STEAP4	1.068961	6.757738	5.777384	5.90E-05	0.001718	1.84786
ZNF385A	1.309329	5.589808	5.773816	5.94E-05	0.001723	1.841738
PPAP2C	-1.13382	5.870544	-5.77169	5.96E-05	0.001724	1.838095
CCL5	1.375507	8.471101	5.767348	6.00E-05	0.001734	1.830637
CNTFR	-1.13031	5.992102	-5.76379	6.04E-05	0.001739	1.824526
PXDNL	-1.37145	8.253578	-5.75536	6.12E-05	0.001745	1.810048
KCNN3	1.057449	5.519732	5.75462	6.13E-05	0.001745	1.808775
RAC2	1.531459	4.945864	5.747344	6.21E-05	0.001754	1.796266
KCNE2	-1.18742	4.701681	-5.743	6.25E-05	0.001765	1.788796
CXCL11	1.386061	5.438793	5.740405	6.28E-05	0.00177	1.784331
SIRPB1	1.200391	4.720491	5.732559	6.36E-05	0.001785	1.770827
HAMP	1.825066	7.344921	5.73015	6.39E-05	0.001785	1.766679
FCGR1A	2.000427	5.092743	5.725697	6.44E-05	0.001792	1.759009
NBEAL2	1.077248	5.677621	5.720247	6.50E-05	0.001803	1.749619
SLC2A3	1.575935	5.484339	5.718546	6.52E-05	0.001805	1.746688

ZNF607	-1.08564	5.156181	-5.71783	6.52E-05	0.001805	1.745459
SYTL3	1.090989	5.689259	5.705287	6.66E-05	0.001838	1.72382
LY96	1.603694	7.182285	5.701033	6.71E-05	0.001847	1.716479
VAMP8	1.301414	9.045046	5.698664	6.74E-05	0.001847	1.712391
GKV1D-39	2.375765	10.39713	5.697853	6.75E-05	0.001847	1.71099
DOCK8	1.722748	6.137774	5.697292	6.76E-05	0.001847	1.710021
COL12A1	2.434341	7.329904	5.692231	6.81E-05	0.001855	1.701281
IGKV1-37	3.027507	7.830395	5.687326	6.87E-05	0.001867	1.692808
NFAM1	1.629327	5.927147	5.684084	6.91E-05	0.001871	1.687204
IGKV1-12	4.004429	6.968936	5.683677	6.91E-05	0.001871	1.6865
GPR84	2.185793	4.490007	5.682267	6.93E-05	0.001871	1.684064
S100P	1.112394	4.86836	5.678733	6.97E-05	0.001878	1.677953
IGLV2-18	3.52401	6.65274	5.67288	7.04E-05	0.001891	1.667831
PLA2G7	3.571714	6.394474	5.671914	7.05E-05	0.001891	1.666159
TRDN	-2.04379	5.925975	-5.66401	7.15E-05	0.001908	1.652477
UCP2	1.675443	6.758605	5.651477	7.30E-05	0.001932	1.63077
TREM2	1.434625	5.206266	5.651455	7.30E-05	0.001932	1.630732
ELMO1	1.227866	6.114487	5.63677	7.49E-05	0.001973	1.605268
MPZL2	1.103656	4.518579	5.630082	7.57E-05	0.00199	1.593662
HRASLS5	-1.24762	6.447396	-5.62483	7.64E-05	0.002002	1.584542
IGLC7	3.125855	6.611704	5.622609	7.67E-05	0.002004	1.580684
CMTM8	1.623668	8.066034	5.610394	7.83E-05	0.002034	1.559457
SELPLG	1.523342	6.271493	5.602761	7.94E-05	0.002055	1.546181
SEMA4D	1.293527	4.719011	5.592907	8.07E-05	0.002078	1.529031
OSBPL3	1.334536	5.321625	5.570742	8.38E-05	0.002144	1.490406
SNX20	1.141401	3.768093	5.566295	8.45E-05	0.002157	1.482648
SIK1	1.316672	5.275187	5.558304	8.56E-05	0.002178	1.468701
RNASE6	1.286425	8.943455	5.54596	8.75E-05	0.002196	1.447139
CLEC4C	1.321968	3.335354	5.544904	8.76E-05	0.002196	1.445294
PDE1A	-1.02448	5.085444	-5.53489	8.91E-05	0.002223	1.427779
EDNRA	1.051424	6.226813	5.532706	8.95E-05	0.002226	1.423964
GPCPD1	1.400525	7.163739	5.532562	8.95E-05	0.002226	1.423712
AIM1	1.283853	5.552217	5.524097	9.08E-05	0.002253	1.408898
HLA-DQB2	1.220593	5.020679	5.522305	9.11E-05	0.002256	1.405761
SRPX2	1.02879	4.511914	5.520191	9.14E-05	0.002258	1.402059
FYB	2.107743	7.827206	5.513412	9.25E-05	0.002276	1.390185
EPHX3	-1.0641	5.136425	-5.51123	9.28E-05	0.002282	1.386366
LCP2	1.897053	7.659078	5.476476	9.86E-05	0.002406	1.325379
FREM1	-1.23564	4.730694	-5.47499	9.88E-05	0.002409	1.322766
STK33	-1.02038	6.321313	-5.46074	0.000101	0.002448	1.297713
DENND1C	1.771413	5.103507	5.457432	0.000102	0.002448	1.291892
MPEG1	1.539453	7.769872	5.456996	0.000102	0.002448	1.291125
PLEK	2.731929	7.004273	5.456391	0.000102	0.002448	1.29006
IGHG2	1.236095	6.650275	5.456267	0.000102	0.002448	1.289843
ABCC3	1.084164	4.997821	5.454633	0.000102	0.00245	1.286965
GKV1D-33	2.886169	7.996611	5.446473	0.000104	0.002478	1.2726
PLAU	2.105663	5.285176	5.441908	0.000105	0.002494	1.264559
OSCAR	1.109798	4.504736	5.430368	0.000107	0.002529	1.244219
GREM1	1.001339	4.210784	5.428245	0.000107	0.002534	1.240474
APOBR	1.606095	5.400087	5.42389	0.000108	0.00255	1.232793
GBP5	2.510172	5.902773	5.421162	0.000108	0.002556	1.227978
PGR	-1.1406	4.29469	-5.41713	0.000109	0.002564	1.220861
CCR1	1.672935	6.156162	5.41439	0.00011	0.002569	1.216026
IGKV3-15	3.19176	8.709664	5.407894	0.000111	0.002585	1.204555
EMP1	1.019963	7.643329	5.394815	0.000114	0.002625	1.18144
LSP1	1.069713	5.213994	5.394788	0.000114	0.002625	1.181393
GKV1D-16	2.907062	8.592747	5.392047	0.000114	0.002634	1.176545
MEST	-1.2417	6.121492	-5.39128	0.000114	0.002634	1.175181

IGKV4-1	3.354419	6.75186	5.369699	0.000119	0.002718	1.136989
TLR8	1.781465	4.77067	5.368297	0.000119	0.002719	1.134505
AREG	1.569283	4.336191	5.367069	0.000119	0.002719	1.13233
FAIM3	1.762555	5.269161	5.364163	0.00012	0.002723	1.12718
AIM2	1.561605	5.046685	5.357205	0.000121	0.002743	1.114846
SIGLEC10	1.858372	5.456673	5.355269	0.000122	0.002746	1.111412
EFNB2	1.257324	5.999921	5.355182	0.000122	0.002746	1.111258
FRRS1L	-1.34566	6.731673	-5.35423	0.000122	0.002747	1.109572
SERPINA1	1.232617	5.502964	5.343383	0.000124	0.002772	1.090323
GLIPR1	1.068396	8.605285	5.337444	0.000126	0.002787	1.079779
AKR1C1	-1.16696	3.356162	-5.33129	0.000127	0.002807	1.06885
VLDLR	-1.65617	5.82127	-5.32544	0.000128	0.002833	1.058452
KIAA1958	-1.20891	6.026338	-5.32141	0.000129	0.002844	1.051293
PF4V1	1.970439	4.846528	5.317337	0.00013	0.002852	1.044045
DOCK2	1.705305	6.075471	5.306591	0.000132	0.002896	1.024925
C10orf107	-1.71407	6.161537	-5.29657	0.000135	0.00293	1.007074
LILRB2	1.445146	5.9213	5.288701	0.000137	0.00295	0.993062
ALOX5	1.523	5.735979	5.28783	0.000137	0.00295	0.991509
GMIP	1.16933	5.464818	5.287554	0.000137	0.00295	0.991017
PTPN6	1.579508	5.624469	5.287259	0.000137	0.00295	0.990491
ARHGDIB	1.125891	6.522107	5.285156	0.000138	0.002957	0.986742
IGHV3-74	2.272538	4.015326	5.277356	0.000139	0.00298	0.972833
SLC14A1	1.235023	4.744063	5.274469	0.00014	0.002992	0.967682
TNFAIP6	2.39682	7.13277	5.270869	0.000141	0.003005	0.961259
NCF4	1.681593	7.070976	5.254952	0.000145	0.003071	0.932835
TNFSF13B	1.452317	6.773279	5.252876	0.000146	0.003079	0.929125
KLRF1	1.526299	4.089055	5.252127	0.000146	0.003079	0.927788
CTSD	1.157226	9.434168	5.231527	0.000151	0.003163	0.890945
MYL3	-1.13489	4.986774	-5.23078	0.000151	0.003164	0.889614
GAPT	1.54484	5.28641	5.229556	0.000152	0.003167	0.887417
HBM	1.758479	6.537067	5.221905	0.000154	0.003174	0.873716
KCNJ15	1.535077	5.701702	5.221533	0.000154	0.003174	0.87305
MMP20	-1.09	10.20841	-5.21775	0.000155	0.003185	0.866272
MYO1F	1.413597	6.295506	5.215083	0.000156	0.003193	0.861495
HNRNPA1L	-1.08127	4.958509	-5.21365	0.000156	0.003195	0.858926
LRRC25	1.518426	4.93349	5.21246	0.000156	0.003195	0.856794
SERPINA3	1.053592	9.956885	5.210378	0.000157	0.003195	0.853063
OLFM2	-1.25651	5.906065	-5.21024	0.000157	0.003195	0.852809
CEACAM1	1.175501	4.370721	5.209247	0.000157	0.003195	0.851035
LAIR2	1.271964	3.366074	5.206171	0.000158	0.003209	0.84552
SP140	1.970219	4.796427	5.197342	0.000161	0.003244	0.829683
TNFRSF1B	1.44789	6.874645	5.191356	0.000162	0.003275	0.818941
MAP1LC3C	-1.03771	3.57884	-5.16761	0.000169	0.003382	0.776286
ZNF114	-1.00018	4.840284	-5.1619	0.000171	0.003405	0.766017
SCARA5	1.152834	4.260236	5.158637	0.000172	0.003417	0.76014
FCAR	1.462152	5.23583	5.156137	0.000173	0.003428	0.755641
NIPSNAP3E	-1.23826	5.803946	-5.15431	0.000173	0.003432	0.752362
SERP2	-1.01584	5.270472	-5.15153	0.000174	0.003442	0.747341
C2	1.184571	5.118365	5.147372	0.000175	0.003462	0.739863
FERMT3	1.427352	5.401816	5.129628	0.000181	0.003532	0.707891
ROBO2	-1.07927	4.836812	-5.1296	0.000181	0.003532	0.707843
ARRB2	1.239923	7.44481	5.126012	0.000182	0.003544	0.701371
CMBL	-1.02494	5.394785	-5.10871	0.000188	0.003627	0.670147
HCAR1	-1.01119	4.873709	-5.1067	0.000188	0.003632	0.666515
TNFAIP3	1.990715	6.408904	5.101429	0.00019	0.003655	0.656998
WNK3	-1.02921	4.413931	-5.09091	0.000194	0.003705	0.637984
NKG7	1.799609	8.673856	5.088901	0.000195	0.00371	0.634356
DAPP1	1.930773	4.870455	5.083508	0.000196	0.003726	0.624602

PP2672	-1.07005	3.380005	-5.07038	0.000201	0.003787	0.600836
PIK3CG	1.374503	6.416058	5.065889	0.000203	0.003806	0.592713
VNN1	1.475678	4.862147	5.064571	0.000203	0.003811	0.590327
GNLY	1.030032	4.636181	5.060174	0.000205	0.003837	0.582361
TRDJ1	-1.17118	6.817683	-5.05174	0.000208	0.003883	0.567076
SLA	1.049278	5.221743	5.033954	0.000215	0.00398	0.534813
KYNU	2.296394	4.923541	5.030406	0.000216	0.003982	0.528373
IGSF6	1.974086	7.449536	5.028226	0.000217	0.003988	0.524414
ARNTL2	1.055922	3.885965	5.026194	0.000218	0.003997	0.520726
PLCB2	1.482845	5.286095	5.01407	0.000222	0.004056	0.498699
WIPF3	-1.28902	5.652532	-5.01034	0.000224	0.004073	0.491921
HLA-DQA1	1.518808	4.539453	5.010287	0.000224	0.004073	0.491822
ACADL	-1.35321	5.751364	-5.00835	0.000225	0.004083	0.488298
GRPR	-1.43093	7.095902	-5.00527	0.000226	0.004102	0.482697
HAPLN1	-1.23705	3.254413	-4.98877	0.000233	0.004185	0.452685
MYO1G	1.712882	4.984073	4.983077	0.000235	0.004218	0.44231
CXorf21	1.663272	5.088468	4.970116	0.000241	0.004284	0.418695
GIMAP7	1.162938	7.932702	4.968563	0.000241	0.004291	0.415862
SORL1	1.329604	5.774635	4.9596	0.000245	0.004348	0.399518
RGS1	2.51152	5.454219	4.953992	0.000248	0.004377	0.389286
CD2	2.119934	5.913279	4.94943	0.00025	0.004402	0.38096
STAB1	1.129945	6.32565	4.947286	0.000251	0.004415	0.377045
C14orf37	-1.23824	6.479438	-4.94657	0.000251	0.004416	0.375747
PILRA	1.821811	6.623281	4.929424	0.000259	0.004529	0.344417
PIM1	1.137767	5.871935	4.913732	0.000266	0.004619	0.315719
HSH2D	2.029816	5.152375	4.905174	0.00027	0.004674	0.300056
MAN2B1	1.015721	7.019119	4.895679	0.000275	0.004722	0.282668
PEG10	-1.19213	8.186741	-4.89508	0.000275	0.004723	0.281572
TBC1D2	1.169756	5.356872	4.893632	0.000276	0.004728	0.278918
DYSF	1.125348	5.059453	4.858005	0.000294	0.004957	0.213572
CLC	2.610631	6.140055	4.839724	0.000304	0.005068	0.179982
HCAR2	3.229947	5.47416	4.839309	0.000305	0.005068	0.179219
APOE	1.436249	4.422127	4.838465	0.000305	0.005068	0.177666
POU2F2	1.055252	4.461386	4.830134	0.00031	0.005114	0.162346
FFAR2	2.418034	4.756423	4.829057	0.00031	0.00512	0.160365
BAZ1A	1.084264	7.125627	4.825063	0.000313	0.005143	0.153015
STAT4	1.245114	5.010967	4.819764	0.000316	0.005173	0.143263
MAP7D2	-1.0865	5.312168	-4.81942	0.000316	0.005173	0.142627
RNASE2	2.113014	6.104304	4.79038	0.000333	0.005385	0.089125
IGJ	2.911459	6.829347	4.783519	0.000337	0.005438	0.07647
SAA1	3.693241	8.868276	4.770663	0.000345	0.005533	0.052741
SIGLEC14	1.813493	6.256237	4.765039	0.000349	0.00558	0.042355
GPNMB	2.350452	6.959953	4.756149	0.000354	0.005646	0.025931
CCR4	1.492362	4.842125	4.737821	0.000366	0.005798	-0.00796
SNX10	1.183165	5.519584	4.735056	0.000368	0.005807	-0.01307
IL1B	3.322331	5.069426	4.731519	0.000371	0.005834	-0.01962
CCL18	2.128362	6.145724	4.715907	0.000381	0.005942	-0.04853
CARD16	1.261684	5.203173	4.70472	0.000389	0.006023	-0.06926
PIM2	2.408677	5.141688	4.69831	0.000394	0.006074	-0.08114
MICAL1	1.086576	6.065318	4.691063	0.000399	0.006137	-0.09458
CCDC69	1.321996	6.386133	4.690802	0.000399	0.006137	-0.09507
SLAMF8	1.99865	6.407796	4.687395	0.000402	0.006165	-0.10139
SULT1B1	1.019494	3.726496	4.685217	0.000403	0.006184	-0.10543
TRAJ31	1.960629	3.546421	4.680813	0.000407	0.006209	-0.11361
ZNF418	-1.15615	4.894966	-4.676	0.00041	0.006253	-0.12254
ABI3	1.086848	5.947924	4.665998	0.000418	0.006332	-0.14112
PTGER4	1.134754	4.666457	4.663727	0.00042	0.006347	-0.14534
HLA-DPB1	1.050723	7.522598	4.662881	0.00042	0.006347	-0.14691

HIST1H3I	1.589774	7.151082	4.657563	0.000424	0.006393	-0.15679
IGHV1-18	4.103758	4.823816	4.655917	0.000426	0.006402	-0.15985
CD80	1.068433	4.100448	4.655531	0.000426	0.006402	-0.16057
HCST	1.095073	8.778317	4.653804	0.000427	0.006406	-0.16378
HLA-DOA	1.064629	6.253882	4.648663	0.000431	0.006437	-0.17334
DPEP2	1.333294	4.633231	4.648163	0.000432	0.006437	-0.17427
PF4	1.463708	5.970017	4.648008	0.000432	0.006437	-0.17456
CNTN1	-1.21686	6.59485	-4.64246	0.000436	0.006448	-0.18488
CD3D	1.797341	6.458651	4.634894	0.000442	0.006483	-0.19896
MYO3A	-1.04796	4.365495	-4.63342	0.000444	0.006495	-0.20171
IGFLR1	1.441071	6.568491	4.628654	0.000447	0.006521	-0.21058
CD14	1.15936	7.386101	4.626274	0.000449	0.006544	-0.21501
TRBV4-1	1.069732	4.117559	4.624322	0.000451	0.006557	-0.21864
PIK3AP1	1.604925	6.393527	4.619131	0.000455	0.006589	-0.22831
NPAS3	-1.0532	4.251478	-4.61111	0.000462	0.006655	-0.24326
NFKBIZ	1.022398	7.423511	4.610536	0.000463	0.006657	-0.24433
MMRN1	1.591405	5.14819	4.608582	0.000464	0.006665	-0.24797
IGKV2-24	2.915464	8.425729	4.60638	0.000466	0.006687	-0.25208
IL7R	1.333274	6.561424	4.60444	0.000468	0.00669	-0.25569
BTG2	1.441074	8.801622	4.600767	0.000471	0.006714	-0.26254
TAGAP	2.229949	6.211343	4.600267	0.000471	0.006714	-0.26347
VWC2	-1.64361	5.488604	-4.57305	0.000496	0.006941	-0.31427
S100A12	1.234734	8.255112	4.56461	0.000503	0.007018	-0.33003
ARHGAP25	1.69305	5.315472	4.56022	0.000508	0.007053	-0.33824
ORM2	1.823661	4.302851	4.557209	0.00051	0.007077	-0.34387
AOAH	1.625917	6.56467	4.555925	0.000512	0.007078	-0.34627
ARHGAP9	1.115973	5.185987	4.555683	0.000512	0.007078	-0.34672
CLGN	-1.22937	4.911086	-4.54572	0.000521	0.007152	-0.36536
MYH11	1.022804	7.531519	4.542026	0.000525	0.00718	-0.37226
SASH3	1.847185	5.586689	4.535895	0.000531	0.007234	-0.38373
ITGAM	1.313752	5.183347	4.528442	0.000538	0.007318	-0.39768
GCH1	1.514945	4.903741	4.508898	0.000558	0.007526	-0.43429
IGHV4-31	3.273372	8.2983	4.500748	0.000567	0.007602	-0.44957
FGR	1.284977	5.130349	4.499471	0.000568	0.007609	-0.45196
CDKL4	-1.44482	3.689643	-4.499	0.000568	0.007609	-0.45284
ITGAL	1.565663	5.375277	4.492368	0.000575	0.00768	-0.46528
TRBJ2-7	1.889873	4.475474	4.483475	0.000585	0.007752	-0.48197
EOMES	1.063923	4.577587	4.481554	0.000587	0.007774	-0.48557
MCOLN3	-1.04889	6.029993	-4.47228	0.000597	0.007858	-0.50299
CPA3	1.632966	3.848957	4.470793	0.000599	0.007866	-0.50577
HLA-B	1.31323	6.755639	4.468873	0.000601	0.007874	-0.50938
ROR1	-1.23107	5.454887	-4.46026	0.000611	0.007983	-0.52556
ADAMDEC1	3.19085	5.70817	4.455507	0.000616	0.008026	-0.53449
WDR72	-1.23952	7.356637	-4.45477	0.000617	0.008031	-0.53588
LY86	1.218377	6.441901	4.448562	0.000624	0.008105	-0.54754
ST8SIA4	1.516967	6.138212	4.437796	0.000637	0.008182	-0.56779
IGHV3-15	3.358018	6.418467	4.411608	0.000669	0.008502	-0.61708
IGLV6-57	3.197803	4.179379	4.405352	0.000677	0.008584	-0.62886
DUSP5	1.005452	4.597564	4.400005	0.000683	0.008648	-0.63894
KIAA1107	-1.18352	6.29781	-4.39765	0.000686	0.008672	-0.64338
NUSAP1	1.119527	4.83235	4.391666	0.000694	0.008739	-0.65465
CDH19	1.064382	6.039161	4.388272	0.000698	0.00878	-0.66105
HBD	1.072616	4.024925	4.386129	0.000701	0.008794	-0.66509
ERBB4	-1.03572	6.291637	-4.38297	0.000705	0.008823	-0.67104
APOC1	1.745116	9.472792	4.382962	0.000705	0.008823	-0.67106
CTSH	1.159671	5.036341	4.366134	0.000728	0.009041	-0.70281
GPR65	1.904445	6.266853	4.362241	0.000733	0.009095	-0.71016
FGFR2	-1.24066	5.591461	-4.3582	0.000739	0.009145	-0.71779

IRF4	2.214475	4.686613	4.354202	0.000744	0.009189	-0.72534
TRAJ21	1.673316	4.763332	4.346414	0.000755	0.009286	-0.74005
MZB1	2.766297	5.783895	4.343353	0.00076	0.009314	-0.74583
P2RX1	1.205125	4.693985	4.330155	0.000779	0.009477	-0.77078
CCL3L3	2.86701	8.268558	4.312333	0.000805	0.009677	-0.80448
PYHIN1	1.026538	5.152551	4.31184	0.000806	0.009679	-0.80542
CYBB	1.415375	8.848436	4.31052	0.000808	0.00969	-0.80791
PMP2	1.006479	8.491253	4.308684	0.000811	0.00971	-0.81139
LRRTM3	-1.53212	4.757906	-4.30757	0.000812	0.009724	-0.8135
NLRC5	1.035612	5.292521	4.297575	0.000828	0.009843	-0.83241
iHV1OR15-	1.471914	4.959054	4.291765	0.000837	0.009881	-0.84342
IKZF1	1.528282	4.824698	4.289908	0.00084	0.009902	-0.84693
CCL4	3.42801	7.910892	4.289829	0.00084	0.009902	-0.84708
TRAF3IP3	1.358088	5.174614	4.282409	0.000852	0.009998	-0.86114
ITGB2	1.128029	5.422903	4.281411	0.000853	0.01001	-0.86303
LPXN	1.22081	6.73434	4.280243	0.000855	0.010019	-0.86524
PLXNC1	1.631956	6.095404	4.277048	0.00086	0.010038	-0.8713
TNFAIP2	1.56009	6.142682	4.276832	0.000861	0.010038	-0.8717
CCRL2	1.142147	3.860063	4.273085	0.000867	0.010083	-0.8788
PARP15	1.469424	3.69922	4.271262	0.00087	0.010112	-0.88226
ATP1B1	-1.04826	7.912208	-4.25362	0.000899	0.01038	-0.91572
PTPRCAP	1.656008	7.350955	4.250347	0.000904	0.010411	-0.92192
IKZF3	1.62217	5.30082	4.237324	0.000927	0.010596	-0.94663
GKV2D-24	3.078053	6.879545	4.233368	0.000934	0.010635	-0.95414
IGHV4-61	2.895613	4.472966	4.210018	0.000976	0.010976	-0.99849
GZMA	1.831752	7.051784	4.203848	0.000987	0.011077	-1.01021
SECTM1	1.325875	6.513857	4.202918	0.000989	0.01109	-1.01198
GPR171	1.114856	5.084224	4.187074	0.001019	0.011344	-1.04211
C10orf55	1.665988	4.125826	4.184965	0.001023	0.011374	-1.04612
NPY1R	-1.10715	6.622877	-4.1762	0.00104	0.01155	-1.0628
CXCL13	3.37175	5.899965	4.174677	0.001043	0.011576	-1.06569
RASAL3	1.178293	5.335783	4.173763	0.001045	0.011586	-1.06743
PCDHB6	-1.00368	4.771398	-4.17211	0.001048	0.011597	-1.07057
IL32	1.15302	4.402058	4.170198	0.001052	0.011625	-1.07421
TXLNB	-1.19617	7.243341	-4.16485	0.001063	0.011721	-1.08438
LILRB4	2.175486	4.438885	4.160758	0.001071	0.011805	-1.09218
CD79A	2.976067	6.227682	4.159188	0.001074	0.011826	-1.09517
MS4A1	3.103787	5.260602	4.151742	0.001089	0.011951	-1.10935
KLHL6	1.691785	5.505533	4.151385	0.00109	0.011951	-1.11003
CXCL3	1.059027	4.56671	4.146531	0.0011	0.01204	-1.11927
PPP1R14A	1.145454	6.534687	4.139039	0.001116	0.012161	-1.13354
CYTIP	1.538924	5.679303	4.138965	0.001116	0.012161	-1.13369
TRAJ22	2.059929	3.999833	4.138695	0.001117	0.012161	-1.1342
VAV1	1.289974	5.783135	4.129054	0.001137	0.01229	-1.15257
GPR37	-1.37433	7.377902	-4.11092	0.001177	0.012622	-1.18715
CNTN6	-1.01437	5.333678	-4.09312	0.001217	0.012904	-1.2211
HLA-DRA	1.024684	10.70676	4.084596	0.001237	0.013046	-1.23737
STAP1	2.030234	4.270592	4.082658	0.001242	0.013086	-1.24107
DUSP2	1.308684	5.480698	4.081207	0.001245	0.0131	-1.24384
IL16	1.027829	5.394402	4.078704	0.001251	0.013132	-1.24862
IGLC2	2.869828	10.63401	4.076208	0.001257	0.013179	-1.25338
SPATC1	1.011702	5.083117	4.065409	0.001283	0.013367	-1.27401
STXBP2	1.231665	6.000536	4.056377	0.001305	0.013537	-1.29126
GKV1D-12	1.49512	3.980343	4.053923	0.001311	0.013592	-1.29595
CTLA4	1.090144	3.369297	4.051925	0.001316	0.013636	-1.29977
CXCL1	1.386737	4.872194	4.050694	0.001319	0.013654	-1.30212
MUCL1	1.170047	3.414976	4.048801	0.001324	0.013671	-1.30574
CSF3	1.028391	4.767438	4.03709	0.001354	0.01392	-1.32812

NPL	1.460942	6.046344	4.035659	0.001358	0.013946	-1.33086
METTL24	-1.09096	5.741023	-4.03439	0.001361	0.013972	-1.33329
C4A	1.445448	6.044716	4.003871	0.001442	0.014553	-1.39166
NFE2	1.057981	3.745641	3.996101	0.001464	0.014705	-1.40653
CXCR2	1.302796	3.510297	3.992899	0.001473	0.014771	-1.41266
GFRA1	-1.01631	6.540272	-3.99098	0.001478	0.0148	-1.41633
TRAJ12	1.811737	2.441708	3.990285	0.00148	0.014808	-1.41766
MCOLN2	1.595231	4.281981	3.986265	0.001492	0.01488	-1.42536
FCN1	1.732134	6.060893	3.981165	0.001506	0.01498	-1.43512
INPP5D	1.385871	5.862189	3.980908	0.001507	0.01498	-1.43562
CXCL5	2.996413	5.612292	3.976634	0.001519	0.015054	-1.4438
IL18RAP	1.036994	4.008444	3.973284	0.001529	0.015109	-1.45022
C4B	1.476551	6.070873	3.972732	0.001531	0.015117	-1.45127
TMC8	1.08419	4.921026	3.965149	0.001553	0.015257	-1.4658
SULT1C4	-1.28251	7.154809	-3.96263	0.00156	0.01532	-1.47061
LILRB3	1.533101	5.503401	3.952644	0.00159	0.015539	-1.48976
CLEC4D	1.536922	3.792484	3.948623	0.001603	0.015634	-1.49746
P2RY13	1.033816	7.163563	3.94649	0.001609	0.015673	-1.50155
NUAK2	1.192797	5.050152	3.941341	0.001625	0.01576	-1.51142
ST6GAL1	1.149681	4.734036	3.940518	0.001628	0.015767	-1.513
MAP4K1	1.57132	4.554917	3.939169	0.001632	0.015767	-1.51558
CD72	1.127026	5.267626	3.938123	0.001635	0.01579	-1.51759
ZNF711	-1.17025	5.713909	-3.93545	0.001643	0.015854	-1.5227
NR4A1	1.474143	4.504595	3.933856	0.001648	0.015894	-1.52577
OTOP1	-1.3029	4.629366	-3.91753	0.001701	0.016188	-1.55707
HEMGN	1.394778	5.319315	3.917228	0.001702	0.016188	-1.55765
TMEM71	1.006682	4.635805	3.916076	0.001705	0.016199	-1.55986
JAK3	1.009789	6.137598	3.905144	0.001741	0.016378	-1.58083
EMR1	1.151378	3.985454	3.900532	0.001757	0.016478	-1.58968
IGLV3-21	2.432519	4.986475	3.897564	0.001767	0.01655	-1.59538
PPEF1	-1.08003	7.094595	-3.89747	0.001767	0.01655	-1.59555
TRAJ4	1.384833	3.961048	3.897169	0.001768	0.01655	-1.59614
RASSF6	1.91685	2.780041	3.896739	0.00177	0.016555	-1.59696
C8orf37	-1.00715	4.909048	-3.89043	0.001791	0.016711	-1.60907
CD3E	1.636693	4.870305	3.889974	0.001793	0.016711	-1.60994
SLC16A6	1.26096	4.699882	3.880188	0.001827	0.016898	-1.62872
CD48	1.673176	6.508123	3.876056	0.001841	0.017015	-1.63666
HOXB2	1.067011	4.374705	3.873654	0.00185	0.017065	-1.64127
TNR	1.040954	4.653405	3.87043	0.001861	0.017118	-1.64746
ANKRD30E	1.088602	2.507261	3.867693	0.001871	0.017167	-1.65271
TRBJ2-1	1.504722	5.289622	3.867256	0.001872	0.017167	-1.65355
TPSB2	1.313512	4.940102	3.859402	0.001901	0.017286	-1.66863
SIGLEC7	1.710015	5.502365	3.857377	0.001908	0.017319	-1.67252
TRAV36DV	-1.37078	3.981743	-3.8569	0.00191	0.017325	-1.67343
KCTD16	1.028967	4.313523	3.853168	0.001924	0.017373	-1.6806
SERPINE1	1.572245	7.454884	3.849524	0.001937	0.01746	-1.6876
RHPN2	-1.1511	5.871856	-3.84148	0.001967	0.017711	-1.70305
SEL1L3	1.637549	5.602951	3.823172	0.002038	0.01807	-1.73823
ZBP1	1.549916	4.169321	3.820731	0.002047	0.018146	-1.74292
CECR1	1.252062	5.648838	3.815101	0.002069	0.018272	-1.75374
GPR174	1.548224	4.282306	3.811348	0.002084	0.018342	-1.76095
LIPN	1.127849	3.178384	3.801878	0.002123	0.018578	-1.77916
MS4A7	1.198725	8.720059	3.79546	0.002149	0.018747	-1.79149
GPR97	1.965327	5.529492	3.783306	0.0022	0.01899	-1.81486
SLAMF6	1.698079	5.258827	3.77799	0.002222	0.01914	-1.82509
FPR2	1.575926	4.673738	3.762796	0.002288	0.019533	-1.85431
GPR137C	-1.00064	5.016319	-3.75855	0.002307	0.019674	-1.86247
HSPA6	1.012941	6.380889	3.757423	0.002312	0.019689	-1.86464

GLUD2	-1.38067	4.457493	-3.7493	0.002348	0.019847	-1.88028
P2RY10	1.73171	5.002832	3.742962	0.002377	0.020062	-1.89246
GKV2D-28	2.852689	8.425015	3.721999	0.002475	0.020659	-1.9328
IGLV7-43	1.818219	3.392105	3.721419	0.002478	0.020659	-1.93391
GALNT6	-1.01567	7.132718	-3.71574	0.002505	0.020795	-1.94484
C3orf62	-1.09451	3.584968	-3.69856	0.00259	0.021249	-1.97791
IGKV1-27	2.885002	6.22105	3.693101	0.002617	0.021425	-1.98842
NUDT11	-1.39165	6.52876	-3.69187	0.002623	0.021428	-1.99078
FHL5	1.146923	3.895498	3.684133	0.002663	0.021671	-2.00568
GZMK	1.780172	5.743361	3.667556	0.002749	0.022179	-2.03759
TRAJ23	1.225248	3.337576	3.665164	0.002762	0.022262	-2.0422
TSPAN8	-1.5511	4.808442	-3.66341	0.002771	0.022318	-2.04557
VPREB3	1.352714	4.499979	3.663401	0.002771	0.022318	-2.04559
TRAJ33	1.81031	4.070555	3.661276	0.002783	0.02239	-2.04968
KIF5C	-1.41785	6.370333	-3.64334	0.002881	0.023006	-2.08423
OSM	1.394883	3.945619	3.640935	0.002894	0.023103	-2.08885
CD3G	1.361462	4.590646	3.633692	0.002935	0.023353	-2.1028
TRAV39	-1.63538	5.136417	-3.6244	0.002988	0.023606	-2.12068
PPAPDC1A	1.448737	5.535237	3.62198	0.003002	0.023687	-2.12535
TRAV41	-1.71309	6.472595	-3.60723	0.003089	0.024098	-2.15376
LGR4	-1.13177	7.496011	-3.60566	0.003098	0.02414	-2.15679
DOC2B	1.197647	5.108593	3.601492	0.003123	0.02425	-2.1648
IL10RA	1.086073	7.754448	3.600888	0.003127	0.02426	-2.16597
TRAV26-2	-1.11814	2.772006	-3.60015	0.003131	0.024272	-2.16738
C3AR1	1.042922	5.411836	3.576198	0.00328	0.025052	-2.21352
C5orf63	-1.2138	6.430051	-3.55151	0.00344	0.026001	-2.26105
FTH1	1.22648	7.402321	3.550843	0.003445	0.026006	-2.26234
KCNA3	1.249036	4.979926	3.546549	0.003473	0.026169	-2.27061
CSF2RA	1.112031	5.551949	3.538091	0.003531	0.02647	-2.2869
CD180	1.586598	6.251145	3.530042	0.003586	0.026745	-2.3024
TRAV1-2	1.001177	3.934603	3.525076	0.003621	0.026957	-2.31196
SCIMP	1.087575	6.461714	3.514989	0.003692	0.027266	-2.33138
OLR1	1.42136	3.852819	3.507916	0.003743	0.027516	-2.345
IGHV6-1	3.02349	6.208668	3.503039	0.003779	0.027636	-2.35439
IGKV6-21	2.460522	4.244086	3.502172	0.003785	0.027641	-2.35606
PIEZO2	-1.10715	7.720562	-3.49867	0.003811	0.027795	-2.36281
C6orf183	-1.38886	5.909903	-3.49223	0.003859	0.028028	-2.3752
IGKV2-40	2.712235	6.658675	3.485572	0.003909	0.028248	-2.38802
IGHJ1	1.374012	6.351837	3.469614	0.004032	0.028872	-2.41874
CMKLR1	1.007055	5.647804	3.446219	0.004219	0.029804	-2.46376
TRAV12-1	1.139507	4.144136	3.435105	0.004311	0.030194	-2.48515
FABP4	1.217279	4.346428	3.430282	0.004351	0.030373	-2.49443
TRAJ47	1.466386	3.399072	3.41751	0.00446	0.030992	-2.51899
SFN	-1.29989	4.448962	-3.41605	0.004473	0.031045	-2.5218
SCN9A	1.209704	5.456825	3.401131	0.004604	0.031655	-2.5505
CD38	1.183811	5.98581	3.393123	0.004676	0.03203	-2.5659
KLRB1	1.362499	6.255421	3.375757	0.004837	0.032822	-2.59928
DERL3	1.623111	5.676983	3.373872	0.004854	0.032866	-2.60291
BTK	1.213592	4.621603	3.361083	0.004977	0.033384	-2.62749
CEACAM3	1.245654	4.692494	3.35937	0.004993	0.033433	-2.63078
LBP	1.969088	4.482094	3.359001	0.004997	0.033445	-2.63149
PRR15	-1.10318	5.566912	-3.3582	0.005004	0.033479	-2.63302
PDZRN4	1.059247	3.869108	3.355884	0.005027	0.033561	-2.63748
SNAP91	-1.10966	5.735588	-3.34882	0.005096	0.033766	-2.65106
MEFV	1.160982	5.00122	3.334462	0.005241	0.034357	-2.67863
SYK	1.252071	5.589209	3.326021	0.005327	0.034764	-2.69484
ACAP1	1.187482	5.895899	3.295345	0.005654	0.036326	-2.75373
TNFSF8	1.068024	5.081622	3.29516	0.005656	0.036327	-2.75408

IGKV3-7	1.558901	4.751734	3.277706	0.005851	0.037265	-2.78757
IL1RN	1.866366	5.032432	3.277279	0.005856	0.037283	-2.78838
SFRP2	1.009151	5.996479	3.275808	0.005873	0.037317	-2.79121
TRAJ6	1.013725	2.726707	3.263021	0.006021	0.037959	-2.81572
CD274	1.049386	5.436955	3.255889	0.006105	0.038306	-2.8294
HCAR3	2.235612	6.27984	3.251122	0.006162	0.038514	-2.83853
CP	1.026002	7.375571	3.249928	0.006176	0.038537	-2.84082
SYDE2	-1.08302	6.520354	-3.24564	0.006228	0.03878	-2.84903
RANBP3L	-1.05216	8.438814	-3.24046	0.006291	0.039106	-2.85897
RIPPLY2	-1.10418	5.6839	-3.21528	0.006606	0.04061	-2.90718
BANK1	1.13186	4.023315	3.199069	0.006818	0.041407	-2.9382
GKV2D-4(	2.533025	6.053551	3.19832	0.006827	0.041412	-2.93964
PSD4	1.202055	4.704665	3.196985	0.006845	0.041478	-2.94219
SPN	1.056538	5.12484	3.195382	0.006867	0.04158	-2.94526
GKV2D-2(	3.491032	4.818497	3.188963	0.006953	0.041979	-2.95753
AOC2	-1.2835	9.167673	-3.18342	0.007028	0.042227	-2.96813
TBX22	-1.23903	4.147995	-3.17647	0.007124	0.042588	-2.98142
PCDH7	-1.05459	7.592682	-3.17332	0.007167	0.042748	-2.98743
IGLV3-25	2.760924	6.345766	3.172469	0.007179	0.042763	-2.98907
PSTPIP2	1.185865	5.777907	3.164925	0.007285	0.043245	-3.00348
GBP1	1.420429	6.089338	3.161248	0.007337	0.043394	-3.01051
ITGA2	-1.01501	7.073265	-3.15549	0.00742	0.043614	-3.02151
GK	1.143488	4.376288	3.147198	0.007541	0.044023	-3.03734
TRAJ35	1.759382	4.330934	3.146233	0.007555	0.044053	-3.03918
EAF2	1.645155	5.732723	3.145252	0.007569	0.044114	-3.04105
CDKL2	-1.05063	4.880145	-3.1207	0.007939	0.045641	-3.0879
SPRR2E	1.05984	2.709244	3.112404	0.008068	0.04623	-3.10372
TRAJ44	1.280427	3.44932	3.107255	0.008149	0.046577	-3.11353
EREG	1.111243	3.754028	3.097856	0.008299	0.047013	-3.13144
TRBV29-1	1.187932	2.887819	3.086903	0.008478	0.047603	-3.1523
IL18	1.347469	5.492242	3.08666	0.008482	0.047603	-3.15276
LAX1	1.738187	4.025083	3.05353	0.009045	0.04966	-3.21579
