# Supplementary material for: Identification of Pulpitis-Related Potential Biomarkers Using Bioinformatics Approach
Source: Comput Math Methods Med. 2021 Sep 29;2021:1808361. doi: 10.1155/2021/1808361 (PMC8495466; doi:10.1155/2021/1808361)
Supplement: Supplementary 2 — Supplementary Table 2: genes in the key functional subset. [file 1808361.f2.pdf]

GPR37
CXCL11
CCL5
CXCL2
C5AR1
CXCL1
CCR4
CCL4
CXCR2
HCAR1
CXCR4
HCAR3
HCAR2
FPR2
ADCY4
C3
CXCL9
S1PR4
P2RY13
CCR7
APLNR
NPY1R
CCL21
CXCL13
CHRM2
CXCR1
CXCL3
FPR3
CXCL5
FPR1
PPBP
GPR183
PF4
CCR1
C3AR1
CXCL10
SAA1
