# Supplementary material for: Identification of Pulpitis-Related Potential Biomarkers Using Bioinformatics Approach
Source: Comput Math Methods Med. 2021 Sep 29;2021:1808361. doi: 10.1155/2021/1808361 (PMC8495466; doi:10.1155/2021/1808361)
Supplement: Supplementary 3 — Supplementary Table 3: pulpitis-related genes from GeneCards. [file 1808361.f3.pdf]

CXCL8
DSPP
IL6
TNF
IL1B
NOS2
BLVRB
ASH1L
CALCRL
CD163
MIR506
IL18
IL1A
SCN8A
CCR6
TRPV1
MMP1
KNG1
TIMP1
SIRT1
TLR4
CCL20
MMP2
MMP3
MMP9
CASP1
TLR2
NOD2
NOD1
CD14
CXCL12
TAC1
CALCA
SOD1
CAT
PDE2A
SOD2
NOS3
PDLIM7
DEFB1
DEFB4A
DEFB104A
DEFB103B
CXCR4
HGF
SPARC
TRPV4
CD36
IL1RN
SOX9
SPP1
SCN10A
BMP7
NPY
AIM2
TRPM2
MTDH
BGLAP

ELANE
F2R
VCAM1
ICAM3
CXCL10
VEGFA
MIR223
AOC3
MAOB
P2RX3
MAPK14
PPARG
TGFB1
ICAM1
MAPK8
FN1
TNC
ENO2
CCL2
GRM5
PTGS2
CD40LG
KLKB1
MMP8
A2M
CD79A
CD8A
IL4
MS4A1
ADCY10
CTSG
SELP
BMP6
COX5A
LTF
SIK2
CHGA
OSM
PTX3
DMP1
PECAM1
DEFA1
KLRG1
DEFA3
IGHE
MIR21
SCN9A
NLRP3
SIRT6
XRCC6
AGER
HMGB1
MMP14
CXCR3
FOXO3
MIR410
MET
MAOA

JUN
WNT5A
ACTN2
POU5F1
AGGF1
FLG
MPPED2
