# Supplementary material for: Identification of Pulpitis-Related Potential Biomarkers Using Bioinformatics Approach
Source: Comput Math Methods Med. 2021 Sep 29;2021:1808361. doi: 10.1155/2021/1808361 (PMC8495466; doi:10.1155/2021/1808361)
Supplement: Supplementary 4 — Supplementary Table 4: genes obtained by the intersection between pulpitis-related genes from GeneCards and genes in the key functional subset in the PPI network. [file 1808361.f4.pdf]

IL6
IL1B
CALCRL
IL18
MMP9
TLR2
CD14
SOD2
CXCR4
IL1RN
SPP1
AIM2
CXCL10
ICAM1
CCL2
PTGS2
CD79A
MS4A1
SELP
OSM
